# Draft genomes of two outcrossing wild rice, *Oryza rufipogon* and *O. longistaminata*, reveal genomic features associated with mating‐system evolution

**DOI:** 10.1002/pld3.232

**Published:** 2020-06-11

**Authors:** Wei Li, Qun‐Jie Zhang, Ting Zhu, Yan Tong, Kui Li, Cong Shi, Yun Zhang, Yun‐Long Liu, Jian‐Jun Jiang, Yuan Liu, En‐Hua Xia, Hui Huang, Li‐Ping Zhang, Dan Zhang, Chao Shi, Wen‐Kai Jiang, You‐Jie Zhao, Shu‐Yan Mao, Jun‐ying Jiao, Ping‐Zhen Xu, Li‐Li Yang, Li‐Zhi Gao

**Affiliations:** ^1^ Institution of Genomics and Bioinformatics South China Agricultural University Guangzhou China; ^2^ Plant Germplasm and Genomics Center Germplasm Bank of Wild Species in Southwestern China Kunming Institute of Botany Chinese Academy of Sciences Kunming China; ^3^ College of Life Science Liaoning Normal University Dalian China; ^4^ University of the Chinese Academy of Sciences Beijing China

**Keywords:** adaptation, genome and transcriptome assemblies, mating system evolution, *O. longistaminata* A. Chev. & Roehr., *Oryza rufipogon* Griff.

## Abstract

*Oryza rufipogon* and *O. longistaminata* are important wild relatives of cultivated rice, harboring a promising source of novel genes for rice breeding programs. Here, we present de novo assembled draft genomes and annotation of *O. rufipogon* and *O. longistaminata*. Our analysis reveals a considerable number of lineage‐specific gene families associated with the self‐incompatibility (SI) and formation of reproductive separation. We show how lineage‐specific expansion or contraction of gene families with functional enrichment of the recognition of pollen, thus enlightening their reproductive diversification. We documented a large number of lineage‐specific gene families enriched in salt stress, antifungal response, and disease resistance. Our comparative analysis further shows a genome‐wide expansion of genes encoding NBS‐LRR proteins in these two outcrossing wild species in contrast to six other selfing rice species. Conserved noncoding sequences (CNSs) in the two wild rice genomes rapidly evolve relative to selfing rice species, resulting in the reduction of genomic variation owing to shifts of mating systems. We find that numerous genes related to these rapidly evolving CNSs are enriched in reproductive structure development, flower development, and postembryonic development, which may associate with SI in *O. rufipogon* and *O. longistaminata*.

## 1 INTRODUCTION

The genus *Oryza* belongs to the grass family and consists of more than 20 wild species and two cultivated species (Khush, 1997; Vaughan, 1994). These rice species have been assigned ten genome types (AA, BB, CC, EE, FF, GG, BBCC, CCDD, HHJJ, and HHKK) (Aggarwal, Brar, & Khush, 1997; Ge, Sang, Lu, & Hong, 1999; Nayar, 1973), representing an enormous gene pool for genetic improvement of modern rice cultivars. The majority of alien genes involved in rice improvement are derived from wild AA‐genome species to broaden gene pool of cultivated rice through introgression lines from the other wild relatives of *Oryza*. This bunch of AA‐genome *Oryza* species comprises two cultivated rice (*O. sativa* and *O. glaberrima*) and six wild relatives (*O. barthii*, *O. longistaminata*, *O. nivara*, *O. rufipogon*, *O. meridionalis*, and *O. glumaepatula*), respectively (Vaughan, Morishima, & Kadowaki, 2003). They span a wide range of global pantropical regions and are disjunctively distributed in Asia, Africa, Australia, and South America (Vaughan, 1994). Phylogenomic studies showed that they have generated extensive AA‐genome diversity from a common AA‐genome ancestor and diverged within 4.8 million years (Myr) (Gao et al., 2019; Zhang et al., 2014; Zhu et al., 2014). There were frequent switches of mating systems between selfing (*O. sativa*, *O. glaberrima*, *O. barthii*, *O. nivara*, *O. meridionalis* and *O. glumaepatula*) and outcrossing species (*O. rufipogon* and *O. longistaminata*). This closely spaced series of recent speciation events has occurred with different life‐history and breeding traits (Oka, 1988; Morishima, Sano, & Oka, 1992), providing an unparalleled system for understanding the gene and genome divergence that determines the wealth of phenotypic diversity and adaptive differences among multiple plant lineages.

*O. rufipogon* Griff., belonging to the genus *Oryza*, is thought to be the wild progenitor of Asian cultivated rice, *O. sativa* (Oka, 1988; Cheng et al., 2003; Fuller et al., 2010; Huang, Chen, et al., 2012; Huang, Kurata, et al., 2012; Khush, 1997). This perennial outcrossing plant species widely grows in diverse habitats of tropical and subtropical regions of Asia and Australia (Morishima et al., 1992). As a result of long historical domestication and improvement, *O. sativa* has experienced a considerable loss of genetic diversity through genome‐wide bottlenecks and artificial selection for domestication traits compared to *O. rufipogon* (Kovach, Sweeney, & McCouch, 2007; Xu et al., 2011). Thus, *O. rufipogon* definitely harbors abundant source of novel alleles that are critical and necessary for rice breeding programs in the future. A large number of alien genes from *O. rufipogon* have successfully been introduced into cultivated rice, generating many environmentally resistant, and high‐yielding varieties (Brar & Ramos, 2007), for example, the application of the “wild‐abortive” allele for the fruitful raising of hybrid rice varieties (Lin & Yuan, 1980). In China, *O. rufipogon* is extensively found in Guangdong, Guangxi, Hainan, Yunnan, Hunan, Jiangxi, Fujian, and Taiwan provinces (Gao, Zhang, Zhou, Ge, & Hong, 1996). Unfortunately, human disturbance has driven this species at the edge of extinction (Gao et al., 1996). Recent decades have witnessed the progress in population and conservation genetic studies using multiple molecular markers to obtain novel insights into the extent, distribution, and dynamics of genetic diversity within and among natural populations of Chinese *O. rufipogon* (Gao, 2004). Despite the release of nuclear genomes of *O. rufipogon* and the seven other AA‐genome *Oryza* species (Du et al., 2017; Goff et al., 2002; IRGSP, 2005; Stein, Yu, Copetti, Zwickl, & Zhang, 2018) the lack of a typical *O. rufipogon* genome may seriously impede its evolutionary and functional genomic studies. In‐depth knowledge of genomic variation and population structure within the species is urgently needed to provide timely information useful for developing appropriate and efficient conservation management of wild rice germplasms.

*O. longistaminata* A. Chev. & Roehr., which is native to Africa, is among the eight AA‐genome species in the genus *Oryza*. This wild rice species possesses highly valued traits to improve cultivated rice, including rhizomatousness for perennial rice breeding program (Glover et al., 2010), strong resistance to diseases and abiotic stresses (Song et al., 1995), and self‐incompatibility (SI) for new procedures to generate seeds of hybrid rice (Ghesquiere, 1986). Recently, Zhang et al. (2015) reported a draft genome assembly of *O. longistaminata* with a relatively short (12.5 kb) contig N50 length. However, deciphering the *O. longistaminata* genome is fundamental to uncover molecular mechanisms that determine these remarkable agronomic traits to enhance rice genetic improvement.

The past decades have witnessed the completion of nuclear genomes of the two cultivated rice subspecies and a number of their relatives (Du et al., 2017; Goff et al., 2002; IRGSP, 2005; Shi et al., 2020; Stein et al., 2018; Wang et al., 2014; Yu et al., 2002; Zhang et al., 2014, 2015, 2016). Here, we sequenced and de novo assembled the two outcrossing wild rice genomes, *O. rufipogon* and *O. longistaminata*. We report the draft genome assembly and annotation of a typical *O. rufipogon* as well as its large transcriptome datasets using Illumina and 454 sequencing platforms. We also present an improved genome assembly and the annotation of *O. longistaminata* as well as large transcriptome datasets using Illumina sequencing platforms. Comparisons of all eight *Oryza* AA‐genomes with well‐defined phylogenetic framework and splitting times may undoubtedly serve as an important model that deserves endeavors for obtaining the full‐genome patterns and multispecies perspective of evolutionary dynamics and genome dissimilarity. Access to the unprecedented data of these *O. rufipogon* and *O. longistaminata* genome sequences will be necessary for mining valuable alleles from this wild rice to boost the future rice breeding programs.

## 2 METHODS

### 2.1 Plant materials

An individual plant of *O. rufipogon* (RUF) was collected from a typical natural population grown in Yuanjiang County, Yunnan Province, China (Gao, Wei, Yang, Hong, & Ge, 2001). Another single plant of *O. longistaminata* (LON), which was originally from Botswana, was introduced from IRRI, Los Banos, the Philippines, and grown in the greenhouse of Kunming Institute of Botany, the Chinese Academy of Sciences. High‐quality genomic DNA was extracted from leaves using a modified CTAB method (Porebski, Bailey, & Baum, 1997). Total RNA was individually isolated from the four tissues of *O. rufipogon* and *O. longistaminata*, including 30‐d‐roots, 30‐d‐shoots, panicles at booting stage and flag leaves at booting stage, using a Water Saturated Phenol method (Zhang et al., 2014).

### 2.2 Genome sequencing on Illumina and 454 platforms

Three short‐insert (180, 300, and 500 bp) paired‐end and six long‐insert (2, 4, 6, 8, 20, and 40 Kb) mate‐pair genomic DNA libraries of *O. rufipogon* were constructed and sequenced using Hiseq2000 platform. A 454 sequencing library was also prepared and sequenced using 454 FLX platform. Three short‐insert (300, 360 and 500 bp) paired‐end and five long‐insert (2, 4, 5, 6 and 8 Kb) mate‐pair genomic DNA libraries of *O. longistaminata* were constructed and sequenced using Hiseq2000 platform.

### 2.3 RNA sequencing on Illumina and 454 platforms and transcriptome de novo assembly

RNA sequencing (RNA‐Seq) libraries were constructed and paired‐end sequenced using the Illumina Hiseq2000 platform to generate RNA‐Seq data for *O. rufipogon*. We constructed the four libraries for 30‐d‐roots, 30‐d‐shoots, panicles at booting stage and flag leaves at booting stage, which were sequenced using Roche 454 platform to yield sequencing reads. To obtain the transcriptome of *O. rufipogon,* we assembled Illumina sequencing reads using Trinity (version r20140717) with default parameters (Grabherr et al., 2011). The transcriptome of *O. rufipogon* was also assembled based on 454 sequencing reads using MIRA (Version 4.0.2) with default parameters (Chevreux et al., 2004). RNA‐Seq libraries were also constructed and paired‐end sequenced using the Illumina Hiseq2000 platform to produce RNA‐Seq data for *O. longistaminata*. To obtain the transcriptome of *O. longistaminata,* we assembled Illumina sequencing reads using Trinity (version r20140717) with default parameters (Grabherr et al., 2011).

### 2.4 Estimation of genome sizes

Nuclear genome sizes of the *O. rufipogon* and *O. longistaminata* plants were estimated using flow cytometry analysis, and *O. sativa* ssp. *japonica* cv. Nipponbare (SAT) was selected as standard with 0.794 pg (~389 Mb) genome size (Cavalier‐Smith, 1985; IRGSP, 2005). The genome sizes were further estimated using *k*‐mer frequencies, which were calculated using the pregraph module implemented in SOAPdenovo (version 1.05) (Li et al., 2010).

### 2.5 Assembly of the O. rufipogon and O. longistaminata genomes

Raw sequencing data were preprocessed using Trimmomatic (version 0.33) (Bolger, Lohse, & Usadel, 2014). SOAPdenovo (Luo et al., 2012) was used to assemble the *O. rufipogon* and *O. longistaminata* genomes. Gaps within scaffolds were filled using GapCloser (version 1.12) (Luo et al., 2012). To solve heterozygous regions in the genome, we finally employed Platanus (v1.2.4) (Kajitani et al., 2014) to assemble these two genomes. All the short insert size libraries were input to assemble subprogram to construct contigs with parameters “‐c 20 ‐k 32.” After obtaining contig sequences, PE reads and MP reads were realigned onto these contigs by Platanus with scaffold subprogram with default parameters to determine the orders of the contigs, remove bubbles and branches, and finally form scaffold sequences. To fill gaps in the assembled scaffolds, GapCloser (version 1.12) (Luo et al., 2012) was adopted with parameters “‐p 25 ‐l 150” using PE reads. Then, the assemblies were masked, and HaploMerger (Huang, Chen, et al., 2012; Huang, Kurata, et al., 2012) was used to further remove redundant sequences.

### 2.6 Prediction of protein‐coding genes and quality validation

Repetitive sequences of the *O. rufipogon* and *O. longistaminata* genome assemblies were masked prior to gene predictions. A combined strategy that integrates ab initio, protein and expressed sequence tag (EST) evidences was adopted to predict protein‐coding genes of *O. rufipogon* and *O. longistaminata*. Augustus (version 3.0.3) (Stanke, Steinkamp, Waack, & Morgenstern, 2004), GlimmerHMM (version 3.0.3) (Majoros, Pertea, & Salzberg, 2004) and GeneMarkHMM (version 3.47) (Lukashin & Borodovsky, 1998) were used to detect the hypothetical gene‐coding regions within genomes. The protein sequences from *O. sativa* ssp. *japonica* cv. *Nipponbare* (SAT), *O. nivara* (NIV), *O. glaberrima* (GLA), *O. barthii* (BAR), *O. glumaepatula* (GLU), *O. meridionalis* (MER), *O. brachyantha*, *Zea mays*, *Sorghum bicolor*, and *Brachypodium distachyon* were aligned to the genome assemblies using GenBlastA (version 1.0.1) (She, Chu, Wang, Pei, & Chen, 2009) and further refined by GeneWise (version 2.2.0) (Birney, Clamp, & Durbin, 2004). To improve the quality of gene predictions, we aligned the assembled transcripts to the genome assembly using PASA (Program to Assemble Spliced Alignments) (Haas et al., 2003) to determine the potential gene structures. We used EVidenceModeler (EVM) (Haas et al., 2008) to combine the ab initio gene predictions, protein alignments and transcription alignments described above into weighted consensus gene structures. To validate the predicted gene models, protein sequences of SAT (v 7.0) genome were downloaded from MSU database (http://rice.plantbiology.msu.edu/), and then, all these peptide sequences were aligned to gene models using BLAT.

### 2.7 Annotation of repeat sequences

RepeatModeler (http://www.repeatmasker.org/RepeatModeler.html), RECON (Bao & Eddy, 2002), and RepeatScout (Price, Jones, & Pevzner, 2005) were used to build de novo repeat elements. The LTR retrotransposons were identified by LTR_STRUC (McCarthy & McDonald, 2003), LTRharvest (Ellinghaus, Kurtz, & Willhoeft, 2008), LTR_FINDER (Xu & Wang, 2007), MGEScan (Lee et al., 2016), and LTR retriever (Ou & Jiang, 2018). Previously annotated transposons were retrieved from the collected *Oryza* RiTE database (Copetti et al., 2015) and Repbase Update (Jurka et al., 2005). To annotate the repeat sequences in these two new genomes all the upper date were used to create a combined library for RepeatMasker (Chen, 2004; Smit, Hubley, & Green, 2016). Simple Sequence Repeats (SSRs) were identified and located using MISA (http://pgrc.ipk‐gatersleben.de/misa/). We combined SSRs from the plus and minus strands and differences caused by reading frames.

### 2.8 Annotation of noncoding RNA genes

The transfer RNA (tRNA) genes, ribosomal RNA (rRNA) genes, small nucleolar RNA (snoRNA) genes, small nuclear RNA (snRNA) genes, and microRNA (miRNA) genes were predicted using de novo and homology search methods. We used tRNAscan‐SE algorithms (version 1.23) (Lowe & Eddy, 1997) with default parameters to identify tRNA genes. The rRNA genes (5S, 18S, and 28S rRNA genes) were predicted using RNAmmer (Lagesen et al., 2007) algorithms with default parameters. The snoRNA genes were annotated using snoScan with the yeast rRNA methylation sites and yeast rRNA sequences provided by the snoScan (Lowe & Eddy, 1999) distribution. The snRNA genes were identified using the INFERNAL (Nawrocki, Kolbe, & Eddy, 2009) software on the Rfam (Griffiths‐Jones et al., 2005) database (release 9.1) with default parameters. We annotated miRNAs in two steps. First, we downloaded the existing rice miRNA entries from miRBase release 18.0 (Kozomara & Griffiths‐Jones, 2011). Then, the conserved miRNAs were identified by mapping all miRBase‐recorded rice miRNA precursor sequences against the assembled *O. rufipogon* and *O. longistaminata* genomes using BLASTN with cutoffs at E‐value <1e^−5^, identity >80%, and query coverage >80%. Second, additional miRNA genes were identified by aligning all miRBase‐recorded grass miRNA precursor sequences against our assembled genomes using BLASTN with cutoffs at E‐value <1e^−5^, identity >60%, and query coverage >60%.

### 2.9 Gene family clustering and evolutionary analyses

OrthoMCL (version 2.0.9) (Li, Stoeckert, & Roos, 2003) was used to identify gene families among *O. sativa* ssp. *japonica*, *O. rufipogo*n, *O. nivara*, *O. barthii*, *O. glaberrima*, *O. glumaepatula*, *O. longistaminata*, *O. meridionalis*, and *O. punctata* (PUN), which were separately downloaded from MSU Rice Genome Annotation Project Database (http://rice.plantbiology.msu.edu) and *Oryza* AA Genomes Database (Zhang et al., 2014). Second, the filtered protein sequences from these nine rice species were compared using all‐versus‐all Blastp with an E‐value of 1E‐5. Finally, gene families among the nine rice species were clustered using a Markov cluster algorithm (MCL) with an inflation parameter of 1.5. According to the presence and absence of genes for a given species, we retrieved and classified species‐specific gene families. An updated version of CAFE (version 3.1) (De Bie, Cristianini, Demuth, & Hahn, 2006) implemented with the likelihood model was used to examine expansions and/or contractions of gene families. Functional enrichment analysis of gene families with the expansion, contraction, and species‐specificity was performed using Fisher's exact test with false discovery rate (FDR) corrections. PFAM domains or gene ontology (GO) terms for each gene used in functional enrichment analyses were directly extracted from the InterProScan entries.

### 2.10 Phylogenomic analyses

The orthologous gene families among *O. sativa* ssp. *japonica*, *O. rufipogo*n, *O. nivara*, *O. barthii*, *O. glaberrima*, *O. glumaepatula*, *O. longistaminata*, *O. meridionalis*, and *O. punctata* were constructed using OrthoMCL (version 2.0.9) (Li et al., 2003). RAxML package (version 8.1.13) (Stamatakis, 2006) was used to resolve phylogenetic relationships among these nine *Oryza* species. Phylogenomic tree was finally constructed using RAxML package (version 8.1.13) (Stamatakis, 2006) using *O. punctata* as outgroup. Divergence times among these species were estimated using the “*mcmctree*” program implemented in the PAML package (Yang, 1997).

### 2.11 *R*‐gene identification and classification

The identification of *R*‐genes within the *O. sativa* ssp. *japonica*, *O. rufipogo*n, *O. nivara*, *O. barthii*, *O. glaberrima*, *O. glumaepatula*, *O. longistaminata*, *O. meridionalis*, and *O. punctata* genomes was performed using a reiterative method (Zhang et al., 2014). Briefly, protein sequences of these rice genomes were first aligned against the raw Hidden Markov Model (HMM) of NB‐ARC family (PF00931) using HMMER (version 3.1b1) (Finn, Clements, & Eddy, 2011) with default parameters, respectively. High‐quality hits with an E‐value of ≤1E‐60 were retrieved and self‐aligned using MUSCLE (version 3.8.31) (Edgar, 2004) to construct each rice species‐specific NBS HMMs, respectively. Based on these specific HMMs, scanning whole *O. sativa* ssp. *japonica*, *O. rufipogo*n, *O. nivara*, *O. barthii*, *O. glaberrima*, *O. glumaepatula*, *O. longistaminata*, *O. meridionalis*, and *O. punctata* proteomes was again conducted, and genes with each rice species‐specific PF00931 domains were defined as *R*‐genes, respectively. The identified *R*‐genes were further classified using TIR domain (PF01582) and LRR domains (PF00560, PF07725, PF12799, PF13306, PF13516, PF13504, and PF13855). These two types of PFAM domains could be detected using HMMER (version 3.1b1) (Finn et al., 2011). CC domains within *R*‐genes were identified using ncoils (Lupas, Van Dyke, & Stock, 1991) with default parameters.

### 2.12 Construction of the orthology synteny map of the eight AA‐genome *Oryza* species

To aid evolutionary analyses, we accurately identified and aligned orthologous genomic regions from the eight assembled AA‐genomes using MERCATOR (Dewey, 2007) and MAVID (Bray & Pachter, 2004). Orthologous sequence alignments were provided with the confirmed phylogenetic relationships (*O. sativa* ssp. *japonica*: 0.002022, *O. rufipogo*n: 0.003527): 0.000373, *O. nivara*: 0.002659): 0.001616, (*O. barthii*: 0.001242, *O. glaberrima*: 0.001793): 0.001899): 0.001268, *O. glumaepatula*: 0.004631): 0.007018, *O. longistaminata*: 0.011113): 0.003953, *O. meridionalis*: 0.014768).

### 2.13 Identification and analysis of conserved noncoding sequences

We identified conserved noncoding sequences (CNSs) using GERP++ software (Cooper et al., 2005). The nucleotide substitution frequency of a CNS was estimated by $nucleotide\_substitution\_frequency=2\sum_{i<j}\frac{d_{\mathrm{ij}}}{n\left(n-1\right)}$, where *d_ij_* is an estimate of the number of nucleotide substitutions per site between DNA sequence *i* and *j*, and *n* is the number of the examined sequences. For each rapidly evolving region in CNSs, nucleotide substitution frequency was subsequently calculated for each species using an average of nucleotide substitution frequency of a species within this genomic region compared to other seven *Oryza* AA‐genome species. We then used the nucleotide substitution frequency of this rapidly evolving region to compare with average nucleotide substitution frequency of total CNSs by Chi‐squared test.

## 3 RESULTS

### 3.1 Genome sequencing, assembly and quality assessment

We sequenced nuclear genomes of two wild rice species: *O. rufipogon* from Asia and *O. longistaminata* from Africa (Table S1). We performed a whole‐genome shotgun sequence (WGS) analysis with the next‐generation sequencing (NGS) Illumina and 454 platforms. This generated raw sequencing datasets of ~174.85 Gb (RUF) and ~176.09 (LON), thus yielding approximately 450.65‐fold and 449.22‐fold coverage, respectively (Table S2). Using two orthogonal methods, we estimated that genome sizes of *O. rufipogon* and *O. longistaminata* are between ~383 and 388 Mb and between ~363 and 392 Mb, respectively (Figures S1 and S2 and Table S3). These genomes were assembled using the clean reads, resulting in final assembles of ~441.41 Mb (RUF) and ~332.08 Mb (LON), respectively (Table 1 and Table S4). The N50 lengths of the assembled RUF contigs and scaffolds were ~18.88 Kb and ~1.94 Mb, respectively (Table 1; Table S4). About 97.62% of the RUF assembly falls into 391 scaffolds larger than 100 Kb in length (Figure S3a and Table S5). The resulting genome assembly was referred to as Oryza_rufipogon_v1.0. The N50 lengths of the assembled LON contigs and scaffolds were ~16.89 Kb and ~1.13 Mb, respectively (Table 1 and Table S4). The contig N50 and scaffold N50 sizes represent ~1.35‐fold and ~3.10‐fold improvement in length from the previously reported (Zhang et al., 2015) ~12.5 Kb and 364 Kb, respectively. About 96.94% of the LON assembly fell into 419 scaffolds larger than 100 Kb in length (Figure S3b and Table S5). The resulting assembled genome was referred to as Oryza_longistaminata_v1.0.

**TABLE 1:** Summary of genome assemblies and annotation of *O. rufipogon* and *O. longistaminata*

	*O. rufipogon*	*O. longistaminata*
Assembly	
Sequencing Depth (×)	450.7	449.2
Estimated genome size (Mb)	388.0	392.0
Assembled sequence length (Mb)	441.41	332.08
Scaffold N50 (bp)	1,935,928	1,133,199
Contig N50 (bp)	18,879	16,885
Annotation	
Number of predicted protein‐coding genes	52,997	40,014
Average gene length (bp)	2,627	2,769
tRNAs	733	636
rRNAs	64	2
snoRNAs	284	247
snRNAs	146	124
miRNAs	271	340
Transposable elements (%)	36.73	30.16

Abbreviation: miRNA, microRNA; rRNA, ribosomal RNA; snoRNA, small nucleolar RNA; snRNA, small nuclear RNA; tRNA, transfer RNA.

To validate the quality of the *O. rufipogon* and *O. longistaminata* genome assemblies, we first aligned all available DNA and protein sequences of RUF and LON from public databases and obtained mapping rates of ~86.94% and 84.50%, and ~64.43% and 66.08%, respectively (Table S6); second, we mapped high‐quality reads of RUF (~51.79 Gb) and LON (~23.17 Gb) to the assembled genome sequences, showing good alignments with average mapping rate of 94.50% and 85.31%, respectively (Table S7); third, we evaluated the quality of WGS assemblies through aligning the assembled RUF and LON genome sequences to the *O. sativa* ssp. *japonica* cv. *Nipponbare* genome. After removing repeat sequences, average mapping rates and sequence similarities were ~83.17% and 66.51％, and ~97.02% and 94.79％, respectively (Table S8). The assembly quality of RUF was additionally confirmed by aligning a total of the five WGS scaffolds with lengths varying from 385 to 680 Kb against the available contigs assembled using SMRT technology. After eliminating repeat sequence‐masked and gap regions, pairwise alignments yielded high sequence similarities of ~99.54% to 99.64% with 100% coverage of sequence length (Figure S4 and Table S9).

### 3.2 Genome annotation

To aid the gene annotation, we de novo assembled the transcriptomes of *O. rufipogon* and *O. longistaminata*. A total of ~29.79 Gb and 223 Mb (Table S10) RNA‐Seq data from RUF were generated on Illumina Hiseq2000 and Roche 454 platform, respectively. We assembled the Illumina data into 109,000 transcripts with a N50 length of 1,193 bp and a total length of ~112 Mb (Table S11). The transcriptome of *O. rufipogon* was also assembled based on 454 sequencing reads, resulting in 33,496 transcripts with a N50 length of 582 bp and a total sequence length of 18,905,412 bp (Table S11). We also assembled RNA‐Seq data (Table S10) from LON, and this process generated 111,105 transcripts with a N50 length of 1,064 bp and a total sequence length of ~74,561,481 bp (Table S11).

In combination with ab initio prediction, protein and public EST alignments, EVM combing and further filtering, we predicted 52,997 and 40,014 protein‐coding genes for RUF and LON, respectively (Figure S5 and Table S12). After the predicted genes were functionally annotated against InterPro, Pfam, and GO protein databases, we aligned protein sequences of *O. sativa* ssp. *japonica* cv. *Nipponbare* and the above‐mentioned RNA‐Seq data of RUF and LON, representing the major tissue types and different developmental stages, to assess the quality of gene prediction. Our results showed that ~89.8% and ~81.6% gene models were supported by transcripts or proteins (identity ≥30% and coverage ≥90%) in RUF and LON, respectively (Figure S6 and Table S13).

**FIGURE 1:**
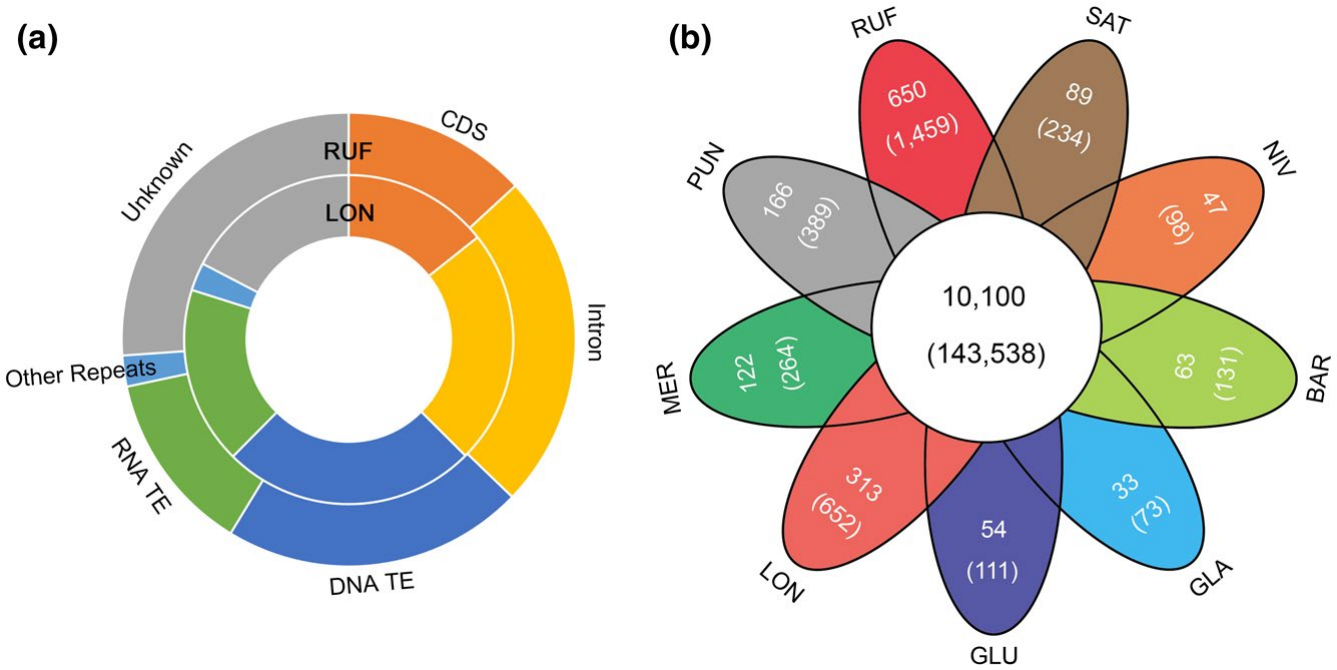
Genome annotation of *O. rufipogon* and *O. longistaminata*. (a) Genome constituents of the annotated genes and repeat sequences; (b) The shared and unique gene families among the eight AA‐genome *Oryza* species using *O. punctata* (BB‐genome) as outgroup

Our annotation of repetitive sequences showed that approximately 45.18% of the RUF genome consists of TEs (Figure 1a and Table S14), slightly lower than that (~50.97%) annotated in the *Nipponbare* genome with the same methods (Figure 1a and Table S14). LTR retrotransposons and *MULE*s were the most abundant TE types, occupying roughly 15.26% and 11.21% of the RUF genome, respectively. The annotation of repetitive sequences revealed that approximately 36.69% of the LON genome consists of TEs (Table S14). LTR retrotransposons and *MULE*s were also the most abundant repeated sequences, occupying approximately 10.66% and 6.53% of the LON genome. We annotated a total of 214,337 SSRs (Table S15) in RUF and 184,773 SSRs (Table S15) in LON, they will provide valuable genetic markers to assist rice breeding programs (Jurka & Pethiyagoda, 1995).

: 6/5/2020, 04:16:53 PM" timestamp="1591354013128">Using de novo and/or homology searches, we annotated ncRNA genes, including tRNA, rRNA, snoRNA, snRNA, and miRNA genes. In total, 733 and 636 tRNA, 64 and 2 rRNA, 284 and 247 snoRNAs, and 146 and 124 snRNAs were characterized in the RUF and LON genomes (Table S16), respectively. Besides, 271 and 340 miRNA genes, belonging to 89 and 84 miRNA families, were identified in the RUF and LON genomes, respectively (Table S16 and Table S17).

### 3.3 Evolutionary dynamics of rice gene families

To investigate evolutionary dynamics of gene families underlying physiological and phenotypic changes and the adaptation of wild rice species, we compared the nine predicted proteomes of *O. sativa*, *O. rufipogon*, *O. nivara*, *O. glaberrima*, *O. barthii*, *O. glumaepatula*, *O. longistaminata*, *O. meridionalis,* and *O. punctata*. A total of 357,284 protein sequences (Table S18) were included in the analysis. Finally, we generated a total of 236,506 orthologous gene families which comprised 304,101 genes (Table S19). This revealed a core set of 143,538 genes belonging to 10,100 gene clusters that were shared among all nine rice species, representing ancestral gene families in AA‐genome *Oryza* species (Figure 1b). Interestingly, 650 (1,459 genes) and 313 (652 genes) gene clusters were found unique to RUF and LON, respectively (Figure 1b). Functional analyses of RUF‐specific genes by both GO terms and PFAM domains revealed the enriched functional categories related to pathogenesis (GO: 0009405, *p* < .001), pollen allergen (PF01357, *p* < .001), stress upregulated Nod 19 (PF07712, *p* <.001), and root cap (PF06830, *p* < .001) (Table S20). Functional analyses of LON‐specific genes further showed functional categories enriched in peroxidase activity (GO: 0004601, *p* < .001), oxidation‐reduction process (GO: 0055114, *p* < .001), and response to oxidative stress (GO: 0006979, *p* < .001) (Table S21). The creation of new gene families in these two wild rice species may have contributed to the observed SI, response to biotic and abiotic stresses, and formation of reproductive separation, which are vital for reproductive success and enhance the abilities of strong adaptation in a remarkably diverse range of habitats in Asia and Africa.

**FIGURE 2:**
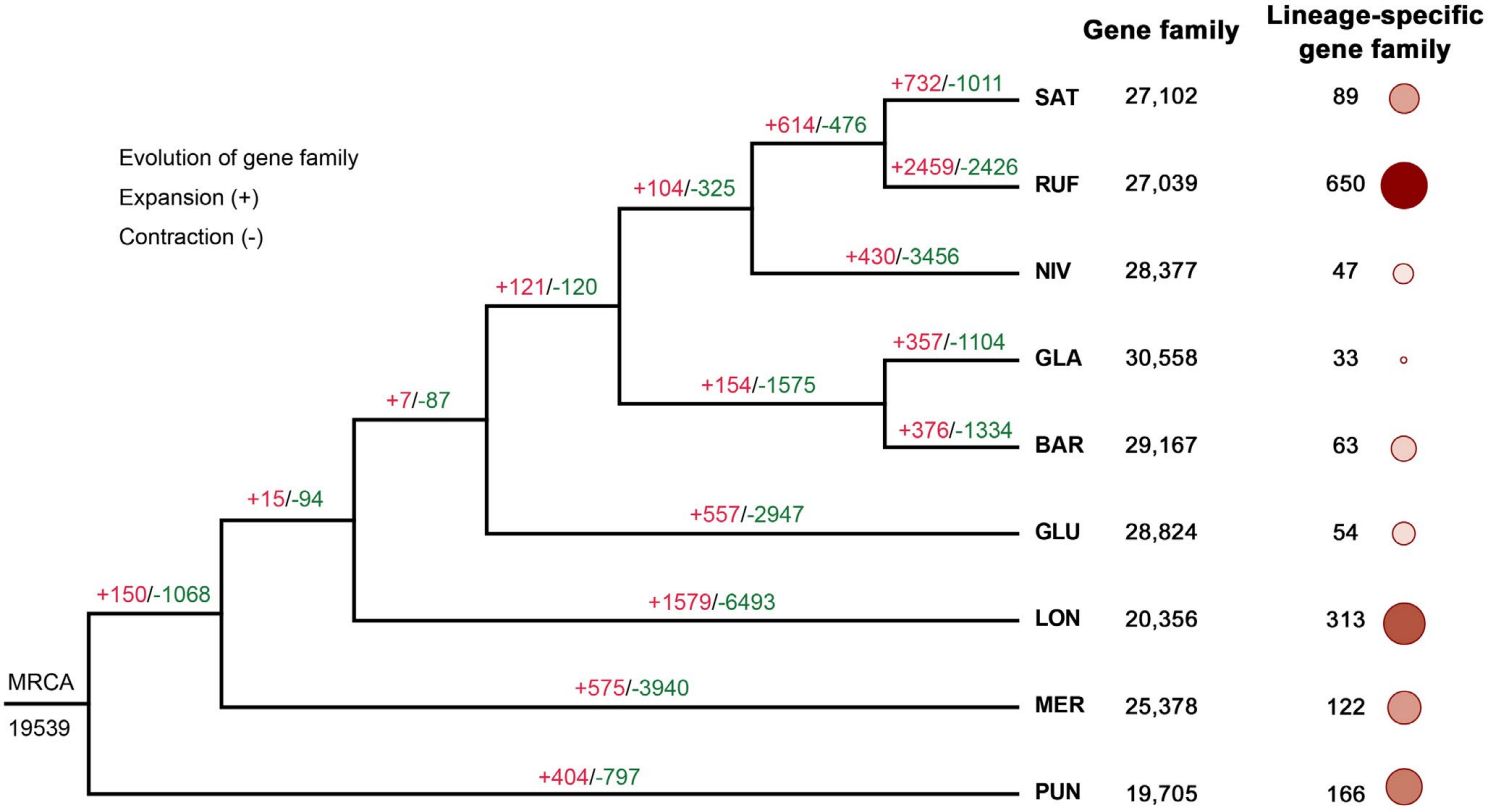
Expansion and contraction of gene families among the eight AA‐genome *Oryza* species using *O. punctata* (BB‐genome) as outgroup

To understand the expansion or contraction of rice gene families causing phenotypic diversification and speciation, we characterized gene families that underwent detectable changes and divergently evolved along different branches with a particular emphasis on those involved in phenotypic traits and environmental adaptation. Our results showed that, of the 19,539 gene families (20,9,968 genes) inferred to exist in the most recent common ancestor of the nine studied rice species, 2,459 (2,426) and 1,579 (6,493) exhibited significant expansions (contractions) (*p* <.001) in the RUF and LON lineages, respectively (Figure 2). Remarkably, functional annotation showed that a large number of genes enriched in functional categories involved in the recognition of pollen (GO: 0048544, *p* < .001), disease resistance, including NB‐ARC domain (PF00931, *p* <.001), Leucine rich repeats (PF13516, PF13855, PF12799, PF00560; *p* < .001) and Leucine rich repeat N‐terminal domain (PF08263, *p* < .001), and salt stress response/antifungal (PF01657; *p* < .001) were significantly amplified in RUF in comparisons with the eight other rice species (Table S22). Compared with the eight other rice species, we surprisingly found that gene families in LON were significantly enriched in a number of functions related to the recognition of pollen (GO: 0048544, *p* < .001), salt stress response/antifungal (PF01657; *p* < .001), and disease resistance, including NB‐ARC domain (PF00931, *p* < .001), Leucine rich repeats (PF13516, PF13855, PF12799, PF00560; *p* < .001), NB‐ARC domain (PF00931; *p* < .001), and Leucine rich repeat N‐terminal domain (PF08263, *p* < .001) (Table S23).

**FIGURE 3:**
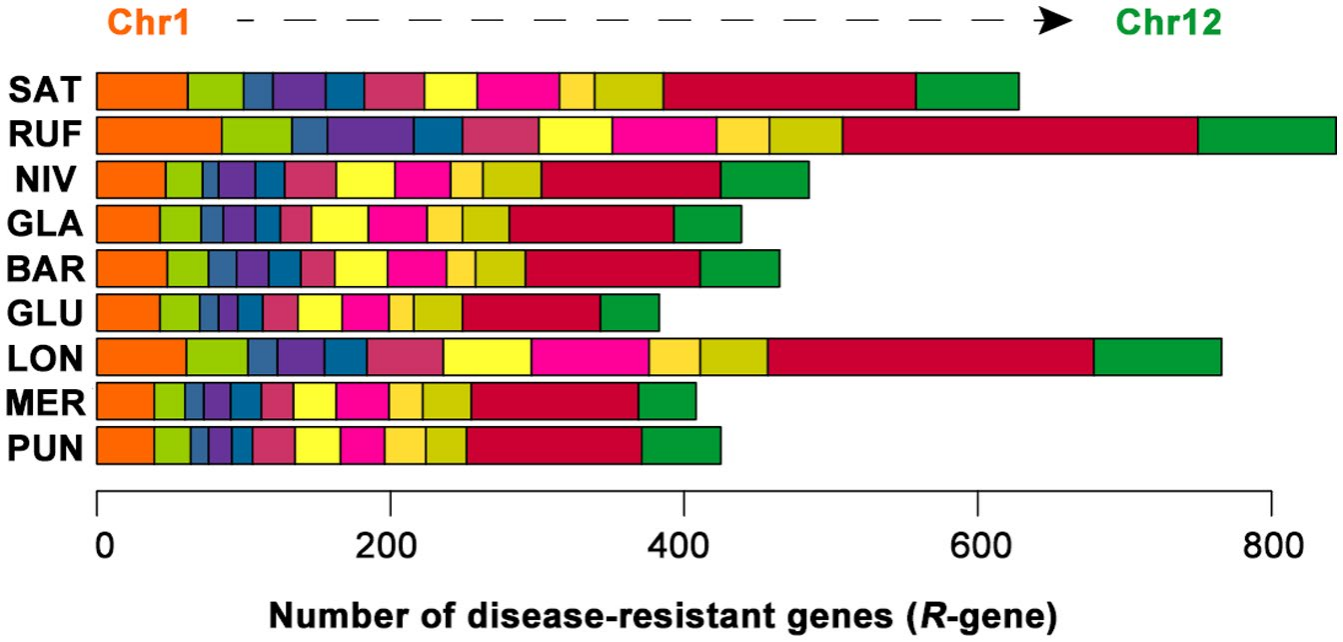
Evolutionary dynamics of the *R*‐genes in the nine *Oryza* genomes

Genome‐wide comparative analysis of the nucleotide‐binding sites with leucine‐rich repeat (NBS‐LRR) genes further showed a remarkable expansion of gene families relevant to an enhanced disease resistance in RUF and LON. In total, we identified 631, 845, 489, 450, 476, 392, 768, 416, and 426 genes encoding NBS‐LRR proteins in SAT, RUF, NIV, GLA, BAR, GLU, LON, MER, and PUN, respectively (Table S24). This amplification in RUF and LON is mainly attributable to an increase in CC‐NBS, CC‐NBS‐LRR, NBS, and NBS‐LRR domains, further supporting that they may have played an important role in biotic resistance and abiotic stresses. We positioned these orthologous *R*‐genes to definite genomic locations across the SAT chromosomes (Figure 3), displaying an almost unequal distribution of the amplified NBS‐encoding genes throughout the entire genome, among which Chromosome 11 harbored the utmost number of *R*‐genes for all these nine rice species. They will evidently offer a large number of candidate loci for further functional genomic studies on disease resistance and rice breeding programs.

### 3.4 Rapid evolution of CNSs

**FIGURE 4:**
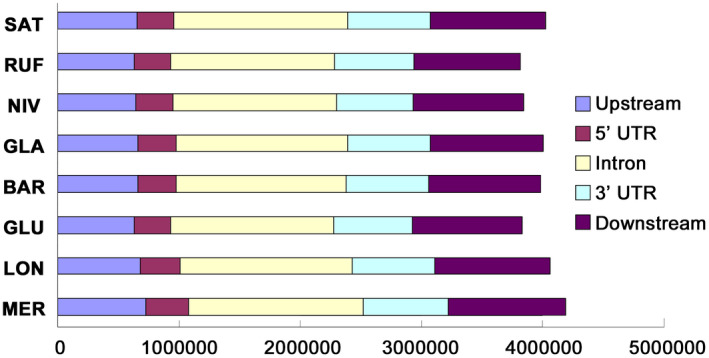
Distribution of the rapidly evolving regions in CNSs across AA‐genome *Oryza* species. Horizontal axis indicates lengths (bp) of the rapid evolving regions. CNS, Conserved noncoding sequences

**FIGURE 5:**
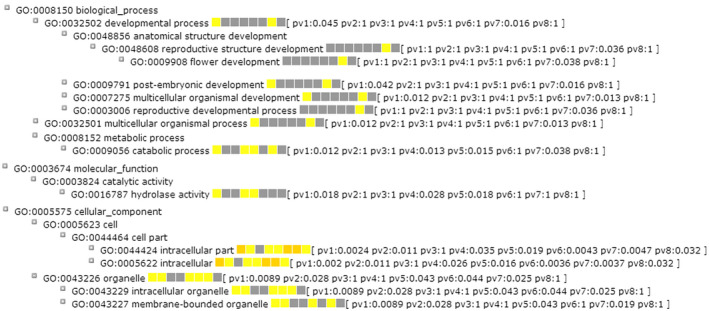
GO annotation of genes related to the rapid evolving regions across AA‐genome *Oryza* species. pv1 represents *p* value of *O. sativa* ssp*. japonica*, pv2 represents *p* value of *O. rufipogo*n, pv3 represents *p* value of *O. nivara*, pv4 represents *p* value of *O. glaberrima*, pv5 represents *p* value of *O. barthii*, pv6 represents *p* value of *O. glumaepatula*, pv7 represents *p* value of *O. longistaminata*, and pv8 represents *p* value of *O. meridionalis*. The yellow‐to‐orange and gray scale represent GO terms that are significance and nonsignificance, respectively. GO, gene ontology

Conserved noncoding sequences are genomic regions showing a reduced mutation frequency of noncoding bases, many of which are regulatory elements that evolve under purifying selection (Haudry et al., 2013). To aid evolutionary analyses of CNSs, we first identified and aligned orthologous genomic regions from the eight AA‐genome assemblies. In total, we obtained 8,742 orthologous genomic segments among the eight AA‐genome *Oryza* species, ranging from 40.02% in RUF to 52.58% in GLA (Table S25). Through this orthologous synteny map as a framework, we identified a total of 67,154 CNSs across the eight AA‐genome *Oryza* species. The total length of these CNSs was 33,258,680 bp, and the length of these CNSs was 495 bp on average. To examine sequence divergence of these CNSs among *Oryza* AA‐genomes, we identified rapidly evolving regions in CNSs by comparing nucleotide substitution frequency between each CNS and the average of all CNSs. The average nucleotide substitution frequency of these regions was 3.46%, which is significantly higher than average level of total CNSs (1.44%, Chi‐squared test,* p* < 2.2e^−16^). A set of 3,123 CNSs was finally identified as rapidly evolving regions (Chi‐squared test,* p* < 0.001). Genomic regions with *p* value < .001 were detected as species‐specific rapidly evolving regions. Results indicated that the distribution of these species‐specific regions that rapidly evolve were associated with upstream 2 Kb regions, 5′UTRs, introns, 3′UTRs, and downstream 2 Kb regions of protein‐coding genes in the eight *Oryza* AA‐genomes (Figure 4). The proportions of the rapidly evolving regions were particularly elevated in introns and downstream regions in comparisons with other genomic regions. Comparisons of species‐specific rapidly evolving regions in CNSs among the eight *Oryza* AA‐genomes showed that nucleotide substitution frequencies of the two outcrossing species, RUF and LON, were higher than those selfing rice species (Table S26). Nucleotide substitution frequency of the rapidly evolving regions within CNSs was 5.68% in *O. longistaminata*, which is significantly higher than the average (3.28%) in *O. sativa* ssp*. japonica*, *O. nivara*, *O. glaberrima*, and *O. barthii* (Chi‐squared test, *p* < 2.2e^−16^). Similarly, nucleotide substitution frequency of the rapidly evolving regions in CNSs of *O. rufipogo*n was 3.79%, which is also significantly higher than its close relatives, such as *O. sativa* ssp. *janonica* (3.06%, Chi‐squared test, *p* < 2.2e^−16^) and *O. nivara* (3.52%, Chi‐squared test, *p* < 2.2e^−16^). The results suggest that a considerable variation of CNSs has been largely reduced as a result of shifts of mating system from outcrossing to selfing. GO of genes in which upstream 2 Kb region, 5′UTR, intron, 3’UTR, and downstream 2 Kb region are associated with the rapidly evolving regions were annotated in each of eight *Oryza* AA‐genomes using AgriGO (Du, Zhou, Ling, Zhang, & Su, 2010). It is intriguing that a number of GO terms related to developmental processes, such as developmental process (GO: 0032502), reproductive structure development (GO: 0048608), flower development (GO: 0009908), postembryonic development (GO: 0009791), and multicellular organismal development (GO: 0007275), were remarkably enriched in *O. longistaminata* (Figure 5). These genomic features may associate with highly valued traits in *O. longistaminata*, such as SI, but functional genomic experiments are further needed.

## 4 DISCUSSION

We report and annotate the two draft genomes of *O. longistaminata* and *O. rufipogon* to supplement the currently existing rich rice genome resources. Compared to the formerly reported *O. longistaminata* genome assembly (Zhang et al., 2015), we produced another contiguous genome assembly that will greatly benefit the research community. Although *O. rufipogon* was recently sequenced (Stein et al., 2018), the generation of the typical *O. rufipogon* genome in this study has the advantage to enhance our understanding of the origin, domestication, and genome evolution of Asian cultivated rice. The genome size of *O. rufipogon* was previously estimated to be ~439 Mb (Uozu et al., 1997), which was seemingly overestimated, compared to the estimations of ~383 and 388 Mb using two orthogonal methods in this study and other representative accessions of the species (Miyabayashi, Nonomura, Morishima, & Kurata, 2007). Probably due to the nature of the typical *O. rufipogon* with a fairly high rate of genomic heterozygosity, we obtained a slightly inflated length of genome assembly (~441.41 Mb), which is larger than the published genome assembly (~339 Mb) (Stein et al., 2018), as divergent haplotypes might be individually represented as separate scaffolds or contigs in the assembled genome.

We detected a considerable number of lineage‐specific gene families associated with the observed SI, response to biotic and abiotic stresses and the formation of reproductive separation in these two outcrossing wild rice species, which may account for their adaptive evolution under the remarkably diverse natural habitats in Asia, Oceania, and Africa. In comparisons with the six other AA‐genome *Oryza* species, we find the rapid evolution of gene families, particularly evidenced by a noticeable fraction showing fast and/or lineage‐specific expansions and contractions with the enrichment of a large number of important functional categories, such as the recognition of pollen in particular; this is obviously related to floral traits of outcrossing and SI observed in *O. rufipogon* and *O. longistaminata*, in sharp contrast with the six other mainly selfing rice species. Besides, a substantial portion of lineage‐specific gene families exhibited significant expansions enriched in a number of functions related to salt stress, antifungal response, and disease resistance. Comparative analysis on whole‐genome level further demonstrates an extraordinary expansion of genes encoding NBS‐LRR proteins in RUF and LON, compared with the six other rice species, including SAT, NIV, GLA, BAR, GLU, and MER, which may associate with their abilities of strong adaptation to remarkably changing environments. One may have concerns about the inflation of the *O. rufipogon* genome assembly that may partially include alternative haplotype to impact our downstream data analysis. In this study, we obtained a genome assembly of ~332.08 Mb for *O. longistaminata*, which was estimated to be between ~363 and 392 Mb using two orthogonal methods. Our above‐described findings indicate that the genome analyses of these two outcrossing wild rice exhibit similar trends of the gene family expansion contrary to the six other predominantly selfing rice species. Thus, the impact of inflated genome assembly of *O. rufipogon* should not be fully excluded but may not considerably influence subsequent data analyses of the gene family evolution.

To avoid mis‐assembly that may affect the downstream data analysis of CNSs, we aligned all eight AA‐genome assemblies and identified stringent orthologous genomic segments across all eight AA‐genome *Oryza* species. Our comparative genomic analyses show a fairly rapid evolution of CNSs in these two outcrossing rice species (RUF and LON) relative to those selfing species, indicating a great reduction of genomic variation within CNSs owning to the switch of mating systems from outcrossing to selfing. Functional enrichment analysis further suggests that genes related to these rapidly evolving CNSs are intriguingly enriched in a large number of important developmental processes, such as reproductive structure development, flower development, and postembryonic development, which may associate with SI in *O. longistaminata*.

A large collection of genomic sequences of Asian cultivated rice and its wild relatives has unquestionably formed a solid foundation to search for novel gene sources from wild rice germplasms (e.g., Du et al., 2017; Goff et al., 2002; IRGSP, 2005; Shi et al., 2020; Stein et al., 2018; Wang et al., 2014; Yu et al., 2002; Zhang et al., 2016; Zhang et al., 2014; Zhang et al., 2015). Future functional experiments should employ advanced genetic tools to study theses disease resistance candidate loci and genomic regulatory elements to determine how they are involved in specific adaptations. We thus expect efforts to generate chromosome‐scale reference genome sequences of wild rice species using long‐read SMRT platform, which would be particularly helpful for comparative genomic analyses of the genus *Oryza*, functional genomic studies, wild rice germplasm utilization towards the future rice breeding programs, and efficient conservation management of the seriously endangered natural populations of these wild rice species.

## CONFLICT OF INTEREST

The authors declare no conflict of interest.

## AUTHORS’ CONTRIBUTIONS

L.G. conceived and designed the study; C.S., C.S., L.Y., S.M., L.Z., and T.Z. contributed to the collection and preparation of the rice samples; H.H. and Y.T. performed flow cytometry experiment; T.Z., J.J., J.J., S.M., and P.X. conducted the library preparation and genome sequencing; W.L., K.L., Q.Z., Y.Z., and Y.Y. performed genome assembly; L.Z. performed RNA preparation; W.L., Y.L., E.X., Y.L., and W.J. assembled and analyzed RNA‐Seq data; W.L., Q.Z., E.X., D. Z., and Y.Z. performed genome annotation; L.G. wrote and revised the manuscript.

## Supporting information

Figs S1‐S6

Tables S1‐S16‐S18‐S19‐S24‐S26

Tables S17‐S20‐S23

## Data Availability

The raw sequence data and genome assemblies reported in this paper have been deposited in the Genome Sequence Archive and Genome Warehouse in BIG Data Center, Beijing Institute of Genomics (BIG), Chinese Academy of Sciences, under accession numbers PRJCA002637.

## References

[pld3232-bib-0001] Aggarwal, R. K. , Brar, D. S. , & Khush, G. S. (1997). Two new genomes in the *Oryza* complex identified on the basis of molecular divergence analysis using total genomic DNA hybridization. Molecular and General Genetics, 254, 1–12. 10.1007/s004380050384 9108284

[pld3232-bib-0002] Bao, Z. , & Eddy, S. R. (2002). Automated *de novo* identification of repeat sequence families in sequenced genomes. Genome Research, 12, 1269–1276. 12176934 10.1101/gr.88502 PMC186642

[pld3232-bib-0003] Birney, E. , Clamp, M. , & Durbin, R. (2004). GeneWise and genomewise. Genome Research, 14, 988–995. 15123596 10.1101/gr.1865504 PMC479130

[pld3232-bib-0004] Bolger, A. M. , Lohse, M. , & Usadel, B. (2014). Trimmomatic: A flexible trimmer for Illumina sequence data. Bioinformatics, 30, 2114–2120. 10.1093/bioinformatics/btu170 24695404 PMC4103590

[pld3232-bib-0005] Brar, D. S. , & Ramos, J. M. (2007). Wild species of Oryza: A rich reservoir of genetic variability for rice improvement. Manila, Philippines: International Rice Research Institute.

[pld3232-bib-0006] Bray, N. , & Pachter, L. (2004). MAVID: Constrained ancestral alignment of multiple sequences. Genome Research, 14, 693–699. 15060012 10.1101/gr.1960404 PMC383315

[pld3232-bib-0007] Cavalier‐Smith, T. (1985). The evolution of genome size, London, UK: Wiley.

[pld3232-bib-0009] Chen, N. (2004) Using RepeatMasker to identify repetitive elements in genomic sequences. Current Protocols in Bioinformatics, 5, 4–10. 11–14.10. 14. 10.1002/0471250953.bi0410s05 18428725

[pld3232-bib-0010] Cheng, C. , Motohashi, R. , Tsuchimoto, S. , Fukuta, Y. , Ohtsubo, H. , & Ohtsubo, E. (2003). Polyphyletic origin of cultivated rice: Based on the interspersion pattern of SINEs. Molecular Biology and Evolution, 20, 67–75. 12519908 10.1093/molbev/msg004

[pld3232-bib-0011] Chevreux, B. , Pfisterer, T. , Drescher, B. , Driesel, A. J. , Muller, W. E. , Wetter, T. , & Suhai, S. (2004). Using the miraEST assembler for reliable and automated mRNA transcript assembly and SNP detection in sequenced ESTs. Genome Research, 14, 1147–1159. 15140833 10.1101/gr.1917404 PMC419793

[pld3232-bib-0012] Cooper, G. M. , Stone, E. A. , Asimenos, G. , Green, E. D. , Batzoglou, S. , & Sidow, A. (2005). Distribution and intensity of constraint in mammalian genomic sequence. Genome Research, 15, 901–913. 15965027 10.1101/gr.3577405 PMC1172034

[pld3232-bib-0013] Copetti, D. , Zhang, J. , El Baidouri, M. , Gao, D. , Wang, J. , Barghini, E. , … Wing, R. A. (2015). RiTE database: A resource database for genus‐wide rice genomics and evolutionary biology. BMC Genomics, 16, 538 10.1186/s12864-015-1762-3 26194356 PMC4508813

[pld3232-bib-0014] De Bie, T. , Cristianini, N. , Demuth, J. P. , & Hahn, M. W. (2006). CAFE: A computational tool for the study of gene family evolution. Bioinformatics, 22, 1269–1271. 10.1093/bioinformatics/btl097 16543274

[pld3232-bib-0015] Dewey, C. N. (2007). Aligning multiple whole genomes with Mercator and MAVID. Methods in Molecular Biology, 395, 221–236. 17993677 10.1007/978-1-59745-514-5_14

[pld3232-bib-0016] Du, H. , Yu, Y. , Ma, Y. , Gao, Q. , Cao, Y. , Chen, Z. , … Liang, C. (2017). Sequencing and de novo assembly of a near complete indica rice genome. Nature Communications, 8, 15324 10.1038/ncomms15324 PMC5418594 28469237

[pld3232-bib-0017] Du, Z. , Zhou, X. , Ling, Y. , Zhang, Z. , & Su, Z. (2010). agriGO: A GO analysis toolkit for the agricultural community. Nucleic Acids Research, 38, W64–W70. 10.1093/nar/gkq310 20435677 PMC2896167

[pld3232-bib-0018] Edgar, R. C. (2004). MUSCLE: Multiple sequence alignment with high accuracy and high throughput. Nucleic Acids Research, 32, 1792–1797. 15034147 10.1093/nar/gkh340 PMC390337

[pld3232-bib-0019] Ellinghaus, D. , Kurtz, S. , & Willhoeft, U. (2008). LTRharvest, an efficient and flexible software for *de novo* detection of LTR retrotransposons. BMC Bioinformatics, 9, 18 10.1186/1471-2105-9-18 18194517 PMC2253517

[pld3232-bib-0020] Finn, R. D. , Clements, J. , & Eddy, S. R. (2011). HMMER web server: Interactive sequence similarity searching. Nucleic Acids Research, 39, W29–37. 10.1093/nar/gkr367 21593126 PMC3125773

[pld3232-bib-0021] Fuller, D. Q. , Sato, Y.‐I. , Castillo, C. , Qin, L. , Weisskopf, A. R. , Kingwell‐Banham, E. J. , … van Etten, J. (2010). Consilience of genetics and archaeobotany in the entangled history of rice. Archaeological & Anthropological Sciences, 2, 115–131. 10.1007/s12520-010-0035-y

[pld3232-bib-0022] Gao, L. (2004). Population structure and conservation genetics of wild rice *Oryza rufipogon* (Poaceae): A region‐wide perspective from microsatellite variation. Molecular Ecology, 13, 1009–1024. 15078440 10.1111/j.1365-294X.2004.02108.x

[pld3232-bib-0023] Gao, L. Z. , Liu, Y. L. , Zhang, D. , Li, W. , Gao, J. , Liu, Y. , … Jiao, J. Y. (2019). Evolution of *Oryza* chloroplast genomes promoted adaptation to diverse ecological habitats. Communications Biology, 2, 278. 31372517 10.1038/s42003-019-0531-2 PMC6659635

[pld3232-bib-0024] Gao, L. , Wei, C. , Yang, Q. , Hong, D. , & Ge, S. (2001). Intra‐population genetic structure of *Oryza rufipogon* Griff. in Yunnan, China. Journal of Plant Research, 114, 107–113.

[pld3232-bib-0025] Gao, L. , Zhang, S. , Zhou, Y. , Ge, S. , & Hong, D. (1996). A survey of the current status of wild rice in China. Chinese Biodiversity, 48, 160–166.

[pld3232-bib-0026] Ge, S. , Sang, T. , Lu, B. R. , & Hong, D. Y. (1999). Phylogeny of rice genomes with emphasis on origins of allotetraploid species. Proceedings of the National Academy of Sciences of the United States of America, 96, 14400–14405. 10588717 10.1073/pnas.96.25.14400 PMC24448

[pld3232-bib-0027] Ghesquiere, A. (1986). Evolution of Oryza longistaminata. Manila, Philippines: International Rice Research Institute.

[pld3232-bib-0028] Glover, J. D. , Reganold, J. P. , Bell, L. W. , Borevitz, J. , Brummer, E. C. , Buckler, E. S. , … Xu, Y. (2010). Agriculture. Increased food and ecosystem security via perennial grains. Science, 328, 1638–1639. 10.1126/science.1188761 20576874

[pld3232-bib-0029] Goff, S. A. , Ricke, D. , Lan, T. H. , Presting, G. , Wang, R. , Dunn, M. , … Briggs, S. (2002). A draft sequence of the rice genome (*Oryza sativa* L. ssp. *japonica*). Science, 296, 92–100. 10.1126/science.1068275 11935018

[pld3232-bib-0030] Grabherr, M. G. , Haas, B. J. , Yassour, M. , Levin, J. Z. , Thompson, D. A. , Amit, I. , … Regev, A. (2011). Full‐length transcriptome assembly from RNA‐Seq data without a reference genome. Nature Biotechnology, 29, 644–652. 10.1038/nbt.1883 PMC3571712 21572440

[pld3232-bib-0031] Griffiths‐Jones, S. , Moxon, S. , Marshall, M. , Khanna, A. , Eddy, S. R. , & Bateman, A. (2005). Rfam: Annotating non‐coding RNAs in complete genomes. Nucleic Acids Research, 33, D121–124. 15608160 10.1093/nar/gki081 PMC540035

[pld3232-bib-0032] Haas, B. J. , Delcher, A. L. , Mount, S. M. , Wortman, J. R. , Smith, R. K. Jr. , Hannick, L. I. , … White, O. (2003). Improving the Arabidopsis genome annotation using maximal transcript alignment assemblies. Nucleic Acids Research, 31, 5654–5666. 14500829 10.1093/nar/gkg770 PMC206470

[pld3232-bib-0033] Haas, B. J. , Salzberg, S. L. , Zhu, W. , Pertea, M. , Allen, J. E. , Orvis, J. , … Wortman, J. R. (2008). Automated eukaryotic gene structure annotation using EVidenceModeler and the Program to Assemble Spliced Alignments. Genome Biology, 9, R7 10.1186/gb-2008-9-1-r7 18190707 PMC2395244

[pld3232-bib-0034] Haudry, A. , Platts, A. E. , Vello, E. , Hoen, D. R. , Leclercq, M. , Williamson, R. J. , … Hazzouri, K. M. (2013). An atlas of over 90,000 conserved noncoding sequences provides insight into crucifer regulatory regions. Nature Genetics, 45, 891–898. 23817568 10.1038/ng.2684

[pld3232-bib-0035] Huang, S. , Chen, Z. , Huang, G. , Yu, T. , Yang, P. , Li, J. , … Xu, A. (2012). HaploMerger: Reconstructing allelic relationships for polymorphic diploid genome assemblies. Genome Research, 22, 1581–1588. 10.1101/gr.133652.111 22555592 PMC3409271

[pld3232-bib-0036] Huang, X. , Kurata, N. , Wei, X. , Wang, Z.‐X. , Wang, A. , Zhao, Q. , … Han, B. (2012). A map of rice genome variation reveals the origin of cultivated rice. Nature, 490, 497–501. 10.1038/nature11532 23034647 PMC7518720

[pld3232-bib-0037] IRGSP . (2005). The map‐based sequence of the rice genome. Nature, 436, 793–800. 10.1038/nature03895 16100779

[pld3232-bib-0038] Jurka, J. , Kapitonov, V. V. , Pavlicek, A. , Klonowski, P. , Kohany, O. , & Walichiewicz, J. (2005). Repbase Update, a database of eukaryotic repetitive elements. Cytogenetic and Genome Research, 110, 462–467. 16093699 10.1159/000084979

[pld3232-bib-0039] Jurka, J. , & Pethiyagoda, C. (1995). Simple repetitive DNA sequences from primates: Compilation and analysis. Journal of Molecular Evolution, 40, 120–126. 7699718 10.1007/BF00167107

[pld3232-bib-0040] Kajitani, R. , Toshimoto, K. , Noguchi, H. , Toyoda, A. , Ogura, Y. , Okuno, M. , … Itoh, T. (2014). Efficient *de novo* assembly of highly heterozygous genomes from whole‐genome shotgun short reads. Genome Research, 24, 1384–1395. 10.1101/gr.170720.113 24755901 PMC4120091

[pld3232-bib-0041] Khush, G. S. (1997). Origin, dispersal, cultivation and variation of rice. Plant Molecular Biology, 35, 25–34. 9291957

[pld3232-bib-0042] Kovach, M. J. , Sweeney, M. T. , & McCouch, S. R. (2007). New insights into the history of rice domestication. Trends in Genetics, 23, 578–587. 17963977 10.1016/j.tig.2007.08.012

[pld3232-bib-0043] Kozomara, A. , & Griffiths‐Jones, S. (2011). miRBase: Integrating microRNA annotation and deep‐sequencing data. Nucleic Acids Research, 39, D152–D157. 10.1093/nar/gkq1027 21037258 PMC3013655

[pld3232-bib-0044] Lagesen, K. , Hallin, P. , Rodland, E. A. , Staerfeldt, H. H. , Rognes, T. , & Ussery, D. W. (2007). RNAmmer: Consistent and rapid annotation of ribosomal RNA genes. Nucleic Acids Research, 35, 3100–3108. 17452365 10.1093/nar/gkm160 PMC1888812

[pld3232-bib-0045] Lee, H. , Lee, M. , Mohammed Ismail, W. , Rho, M. , Fox, G. C. , Oh, S. , & Tang, H. (2016). MGEScan: A Galaxy‐based system for identifying retrotransposons in genomes. Bioinformatics, 32, 2502–2504. 10.1093/bioinformatics/btw157 27153595

[pld3232-bib-0046] Li, L. , Stoeckert, C. J. Jr , & Roos, D. S. (2003). OrthoMCL: Identification of ortholog groups for eukaryotic genomes. Genome Research, 13, 2178–2189. 12952885 10.1101/gr.1224503 PMC403725

[pld3232-bib-0047] Li, R. , Zhu, H. , Ruan, J. , Qian, W. , Fang, X. , Shi, Z. , … Wang, J. (2010). *De novo* assembly of human genomes with massively parallel short read sequencing. Genome Research, 20, 265–272. 20019144 10.1101/gr.097261.109 PMC2813482

[pld3232-bib-0048] Lin, S. C. , & Yuan, L. P. (1980 Hybrid rice breeding in China In Innovative approaches to rice breeding. Selected papers from the 1979 International Rice Research Conference.

[pld3232-bib-0049] Lowe, T. M. , & Eddy, S. R. (1997). tRNAscan‐SE: A program for improved detection of transfer RNA genes in genomic sequence. Nucleic Acids Research, 25, 955–964. 9023104 10.1093/nar/25.5.955 PMC146525

[pld3232-bib-0050] Lowe, T. M. , & Eddy, S. R. (1999). A computational screen for methylation guide snoRNAs in yeast. Science, 283, 1168–1171. 10.1126/science.283.5405.1168 10024243

[pld3232-bib-0051] Lukashin, A. V. , & Borodovsky, M. (1998). GeneMark.hmm: New solutions for gene finding. Nucleic Acids Research, 26, 1107–1115. 9461475 10.1093/nar/26.4.1107 PMC147337

[pld3232-bib-0052] Luo, R. , Liu, B. , Xie, Y. , Li, Z. , Huang, W. , Yuan, J. , … Wang, J. (2012). SOAPdenovo2: An empirically improved memory‐efficient short‐read de novo assembler. Gigascience, 1, 18 10.1186/2047-217X-1-18 23587118 PMC3626529

[pld3232-bib-0053] Lupas, A. , Van Dyke, M. , & Stock, J. (1991). Predicting coiled coils from protein sequences. Science, 252, 1162–1164. 10.1126/science.252.5009.1162 2031185

[pld3232-bib-0054] Majoros, W. H. , Pertea, M. , & Salzberg, S. L. (2004). TigrScan and GlimmerHMM: Two open source *ab initio* eukaryotic gene‐finders. Bioinformatics, 20, 2878–2879. 10.1093/bioinformatics/bth315 15145805

[pld3232-bib-0055] McCarthy, E. M. , & McDonald, J. F. (2003). LTR_STRUC: A novel search and identification program for LTR retrotransposons. Bioinformatics, 19, 362–367. 10.1093/bioinformatics/btf878 12584121

[pld3232-bib-0081] Miyabayashi, T. , Nonomura, K. I. , Morishima, H. , & Kurata N. (2007). Genome Size of Twenty Wild Species of Oryza Determined by Flow Cytometric and Chromosome Analyses. Breeding Science, 57(1), 73–78. 10.1270/jsbbs.57.73

[pld3232-bib-0056] Morishima, H. , Sano, Y. , & Oka, H. I. (1992). Evolutionary studies in cultivated rice and its wild relatives. Taxon, 8, 135–184.

[pld3232-bib-0057] Nawrocki, E. P. , Kolbe, D. L. , & Eddy, S. R. (2009). Infernal 1.0: Inference of RNA alignments. Bioinformatics, 25, 1335 10.1093/bioinformatics/btp157 19307242 PMC2732312

[pld3232-bib-0058] Nayar, N. M. (1973). Origin and cytogenetics of rice. Advances in Genetics, 17, 153–292.

[pld3232-bib-0008] Oka, H. I. (1988). Origin of cultivated rice, Amsterdam: Japan Science Social Press.

[pld3232-bib-0059] Ou, S. , & Jiang, N. (2018). LTR_retriever: A highly accurate and sensitive program for identification of long terminal repeat retrotransposons. Plant Physiology, 176, 1410–1422. 10.1104/pp.17.01310 29233850 PMC5813529

[pld3232-bib-0060] Porebski, S. , Bailey, L. G. , & Baum, B. R. (1997). Modification of a CTAB DNA extraction protocol for plants containing high polysaccharide and polyphenol components. Plant Molecular Biology Reporter, 15, 8–15.

[pld3232-bib-0061] Price, A. L. , Jones, N. C. , & Pevzner, P. A. (2005). *De novo* identification of repeat families in large genomes. Bioinformatics, 21(Suppl 1), i351–i358. 10.1093/bioinformatics/bti1018 15961478

[pld3232-bib-0062] She, R. , Chu, J. S. , Wang, K. , Pei, J. , & Chen, N. (2009). GenBlastA: Enabling BLAST to identify homologous gene sequences. Genome Research, 19, 143–149. 18838612 10.1101/gr.082081.108 PMC2612959

[pld3232-bib-0063] Shi, C. , Li, W. , Zhang, Q. J. , Zhang, Y. , Tong, Y. , Li, K. , … Gao, L. Z. (2020). The draft genome sequence of an upland wild rice species, *Oryza Granulata* . Scientific Data, 7, 131 10.1038/s41597-020-0470-2 32350267 PMC7190833

[pld3232-bib-0064] Smit, A. , Hubley, R. , & Green, P. (2016) RepeatMasker Open‐4.0. 2015. Google Scholar.

[pld3232-bib-0065] Song, W.‐Y. , Wang, G.‐L. , Chen, L.‐L. , Kim, H.‐S. , Pi, L.‐Y. , Holsten, T. , … Ronald, P. (1995). A receptor kinase‐like protein encoded by the rice disease resistance gene, *Xa21* . Science, 270, 1804–1806. 10.1126/science.270.5243.1804 8525370

[pld3232-bib-0066] Stamatakis, A. (2006). RAxML‐VI‐HPC: Maximum likelihood‐based phylogenetic analyses with thousands of taxa and mixed models. Bioinformatics, 22, 2688–2690. 10.1093/bioinformatics/btl446 16928733

[pld3232-bib-0067] Stanke, M. , Steinkamp, R. , Waack, S. , & Morgenstern, B. (2004). AUGUSTUS: A web server for gene finding in eukaryotes. Nucleic Acids Research, 32, W309–W312. 10.1093/nar/gkh379 15215400 PMC441517

[pld3232-bib-0068] Stein, J. C. , Yu, Y. , Copetti, D. , Zwickl, D. J. , & Zhang, L. (2018). Genomes of 13 domesticated and wild rice relatives highlight genetic conservation, turnover and innovation across the genus *Oryza* . Nature Genetics, 50, 285–296. 29358651 10.1038/s41588-018-0040-0

[pld3232-bib-0080] Uozu, S. , Ikehashi, H. , Ohmido, N. , Ohtsubo, H. , Ohtsubo, E. , & Fukui, K. (1997). Repetitive sequences: Cause for variation in genome size and chromosome morphology in the genus Oryza. Plant Molecular Biology, 35(6), 791–799. 10.1023/a:1005823124989 9426599

[pld3232-bib-0069] Vaughan, D. A. (1994) The wild relatives of rice: a genetic resources handbook.

[pld3232-bib-0070] Vaughan, D. A. , Morishima, H. , & Kadowaki, K. (2003). Diversity in the *Oryza* genus. Current Opinion in Plant Biology, 6, 139–146. 12667870 10.1016/s1369-5266(03)00009-8

[pld3232-bib-0071] Wang, M. , Yu, Y. , Haberer, G. , Marri, P. R. , Fan, C. , Goicoechea, J. L. , … Wing, R. A. (2014). The genome sequence of African rice (*Oryza glaberrima*) and evidence for independent domestication. Nature Genetics, 46, 982–988. 25064006 10.1038/ng.3044 PMC7036042

[pld3232-bib-0072] Xu, X. , Liu, X. , Ge, S. , Jensen, J. D. , Hu, F. , Li, X. , … Wang, W. (2011). Resequencing 50 accessions of cultivated and wild rice yields markers for identifying agronomically important genes. Nature Biotechnology, 30, 105–111. 10.1038/nbt.2050 22158310

[pld3232-bib-0073] Xu, Z. , & Wang, H. (2007). LTR_FINDER: An efficient tool for the prediction of full‐length LTR retrotransposons. Nucleic Acids Research, 35, W265–W268. 10.1093/nar/gkm286 17485477 PMC1933203

[pld3232-bib-0074] Yang, Z. (1997). PAML: A program package for phylogenetic analysis by maximum likelihood. Computer Applications in the Biosciences, 13, 555–556. 9367129 10.1093/bioinformatics/13.5.555

[pld3232-bib-0075] Yu, J. , Hu, S. , Wang, J. , Wong, G. K. , Li, S. , Liu, B. , … Yang, H. (2002). A draft sequence of the rice genome (Oryza sativa L. ssp. indica). Science, 296, 79–92. 10.1126/science.1068037 11935017

[pld3232-bib-0076] Zhang, J. , Chen, L. L. , Xing, F. , Kudrna, D. A. , Yao, W. , Copetti, D. , … Xie, W. (2016). Extensive sequence divergence between the reference genomes of two elite *indica* rice varieties Zhenshan 97 and Minghui 63. Proceedings of the National Academy of Sciences of the United States of America, 113, E5163–5171. 27535938 10.1073/pnas.1611012113 PMC5024649

[pld3232-bib-0077] Zhang, Q. J. , Zhu, T. , Xia, E. H. , Shi, C. , Liu, Y. L. , Zhang, Y. , … Gao, L. Z. (2014). Rapid diversification of five *Oryza* AA genomes associated with rice adaptation. Proceedings of the National Academy of Sciences of the United States of America, 111, E4954–E4962. 25368197 10.1073/pnas.1418307111 PMC4246335

[pld3232-bib-0078] Zhang, Y. , Zhang, S. , Liu, H. , Fu, B. , Li, L. , Xie, M. , … Hu, F. (2015). Genome and comparative transcriptomics of African wild rice *Oryza longistaminata* provide insights into molecular mechanism of rhizomatousness and self‐incompatibility. Molecular Plant, 8, 1683–1686. 10.1016/j.molp.2015.08.006 26358679

[pld3232-bib-0079] Zhu, T. , Xu, P. Z. , Liu, J. P. , Peng, S. , Mo, X. C. , & Gao, L. Z. (2014). Phylogenetic relationships and genome divergence among the AA‐genome species of the genus *Oryza* as revealed by 53 nuclear genes and 16 intergenic regions. Molecular Phylogenetics and Evolution, 70, 348–361. 10.1016/j.ympev.2013.10.008 24148990

