# Supplementary material for: Draft genomes of two outcrossing wild rice, Oryza rufipogon and O. longistaminata, reveal genomic features associated with mating‐system evolution
Source: Plant Direct. 2020 Jun 11;4(6):e00232. doi: 10.1002/pld3.232 (PMC7287411; doi:10.1002/pld3.232)
Supplement: Supplementary file 1 — Figs S1‐S6 [file PLD3-4-e00232-s001.doc]

**Supplementary Figures**


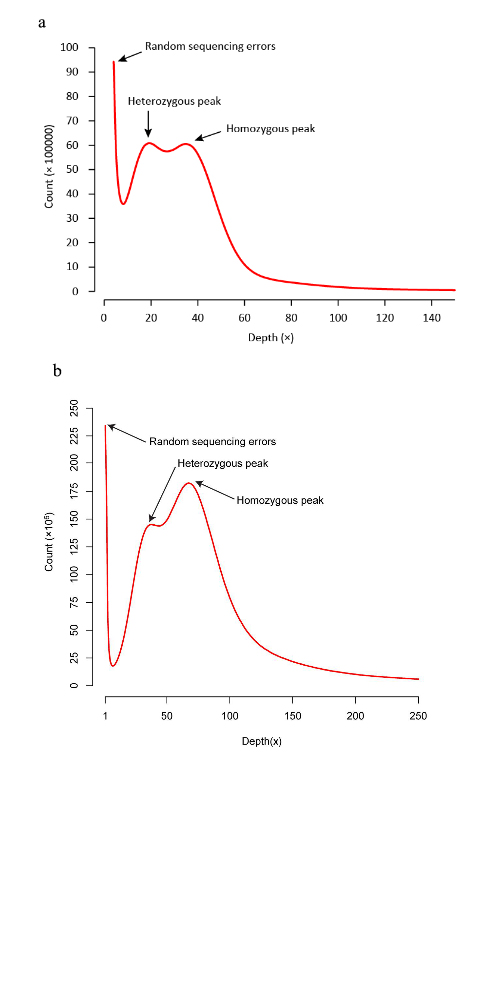


**Supplementary Fig. 1.** **The 17-mer distribution of sequencing reads from *Oryza rufipogon* and *O*. *longistaminata*.** (**a**) The 17-mer distribution of *O. rufipogon*; (**b**) The 17-*mer* distribution of *O. longistaminata*. The occurrence of 17-mer was calculated using GCE (version 1.0.0) based on the sequencing data from short insert size libraries (insert size ≤ 500 bp) of *O. rufipogon*. The sharp peak on the left with low depth represents the essentially random sequencing errors. The middle and right peaks indicate the heterozygous and homozygous peaks, respectively.


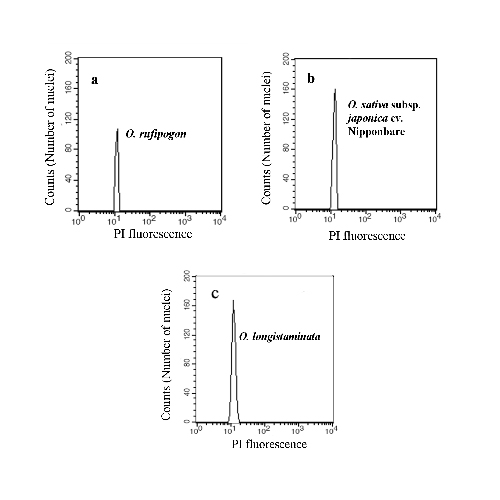


**Supplementary Fig. 2. Cytogram of fluorescence intensity of *O. rufipogon* in comparison with *O. sativa* ssp. *japonica.* cv. Nipponbare nuclei isolated with an improved Otto buffer.** Coefficient of variation values (CVs): (**a**) 3.36%; (**b**) 3.89%; (**c**) 4.07%. X: relative fluorescence; Y: number of nuclei.


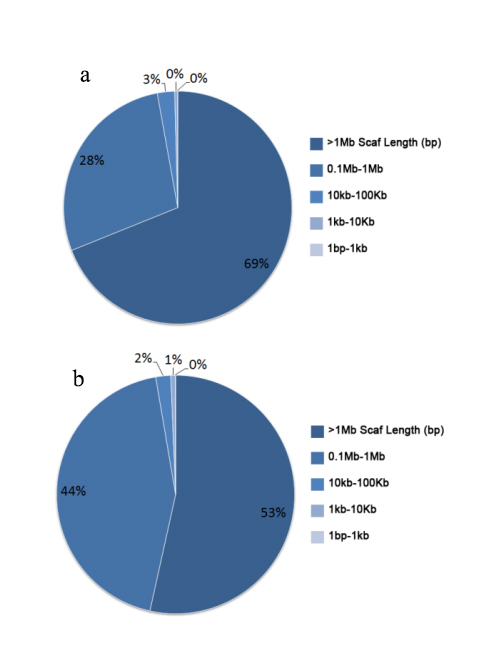


**Supplementary Fig. 3. Length constitutions of scaffolds for the assembled genomes.** (**a**) Length constitutions of *O.* *rufipogon* genome; (**b**) Length constitutions of *O.* *longistaminata* genome.


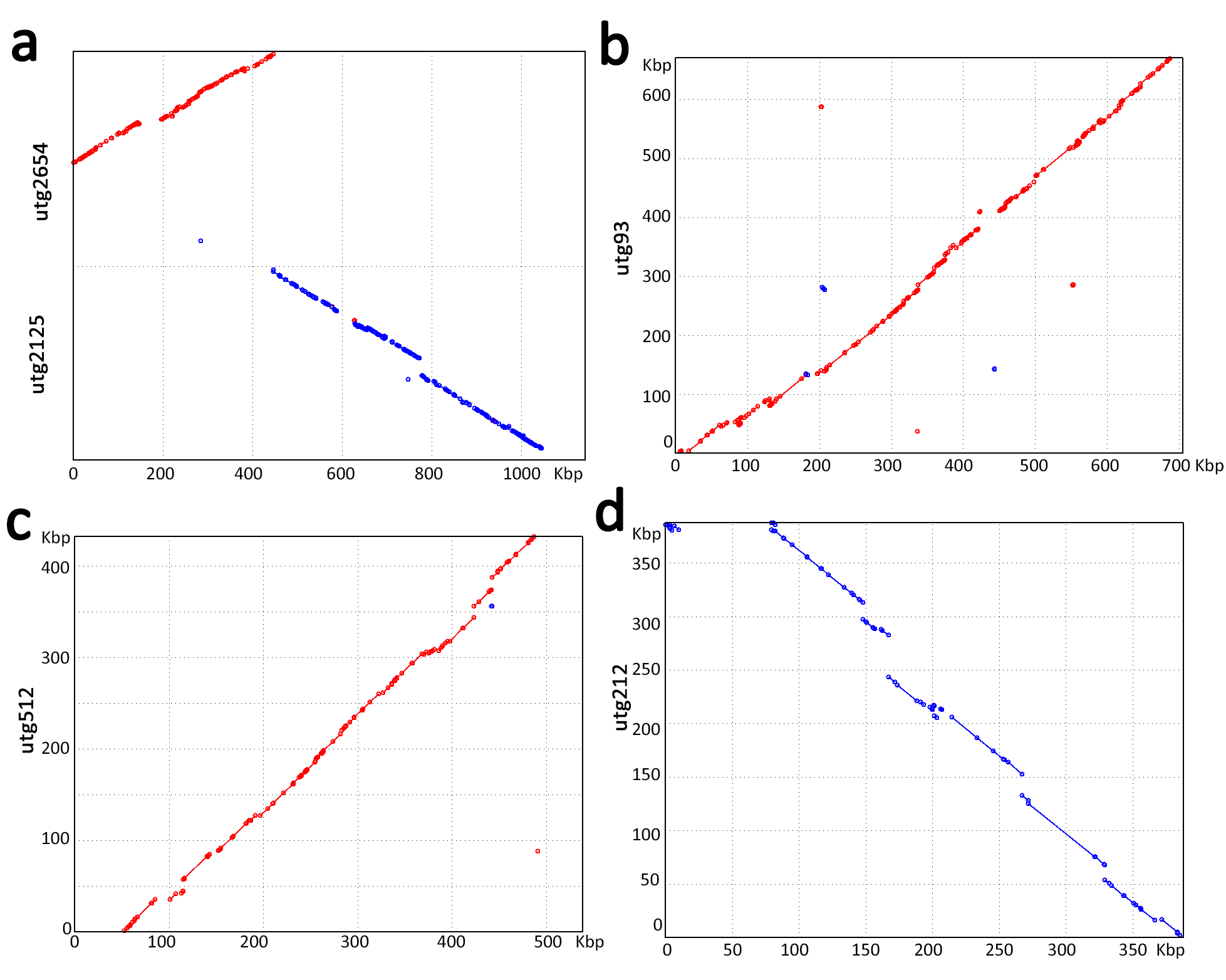


**Supplementary Fig. 4. Examples of scatter plot display of the Illumina assembled scaffolds that make alignments with the corresponding SMRT based contigs.** (**a**) Scaffold149 alignment, (**b**) scaffold225 alignment, (**c**) scaffold281 alignment, and (**d**) scaffold334 alignment.


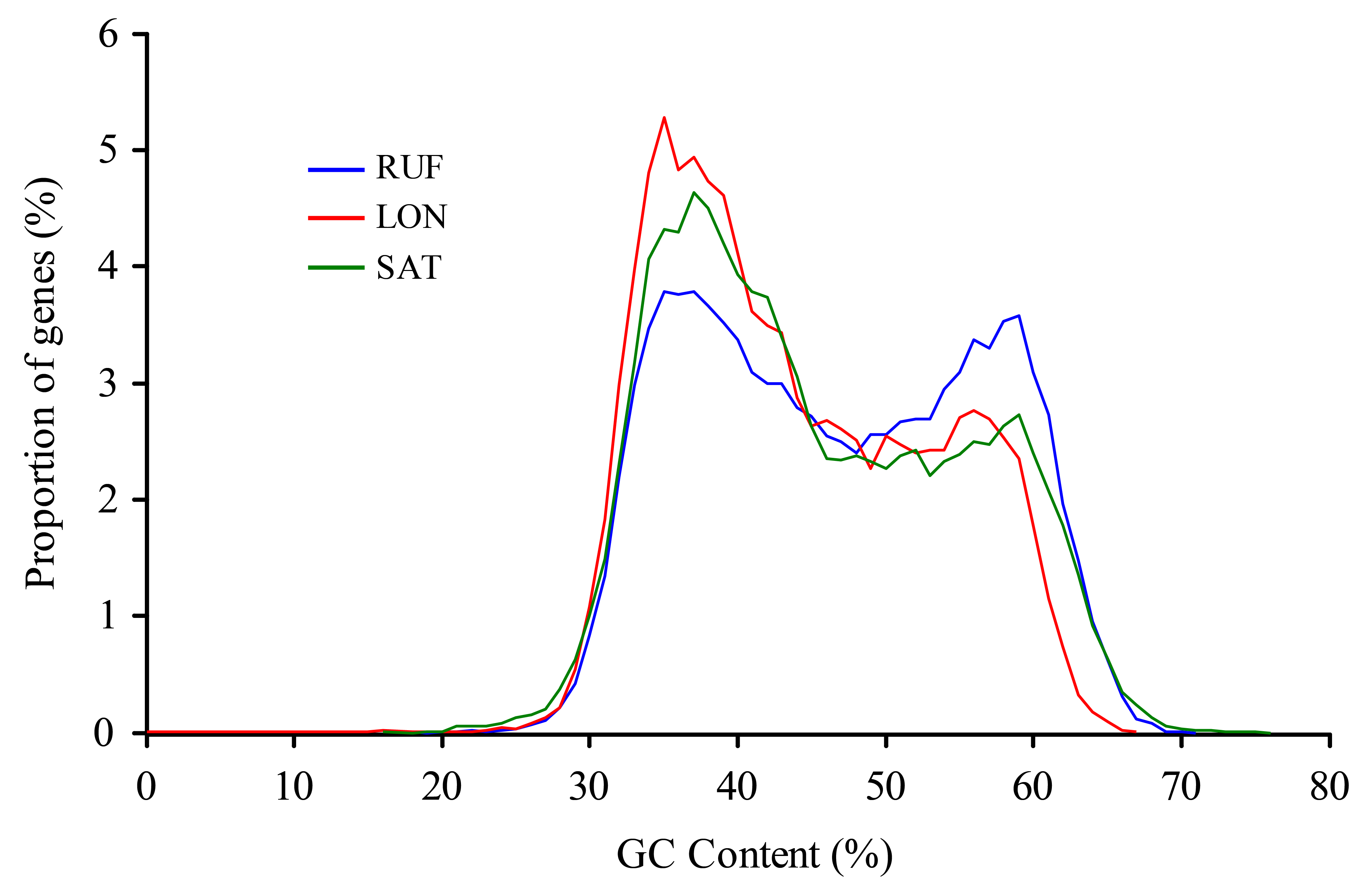


**Supplementary Fig. 5. GC distribution of the coding sequences (CDs) among *O. rufipogon*, *O. longistaminata* and *O. sativa***.


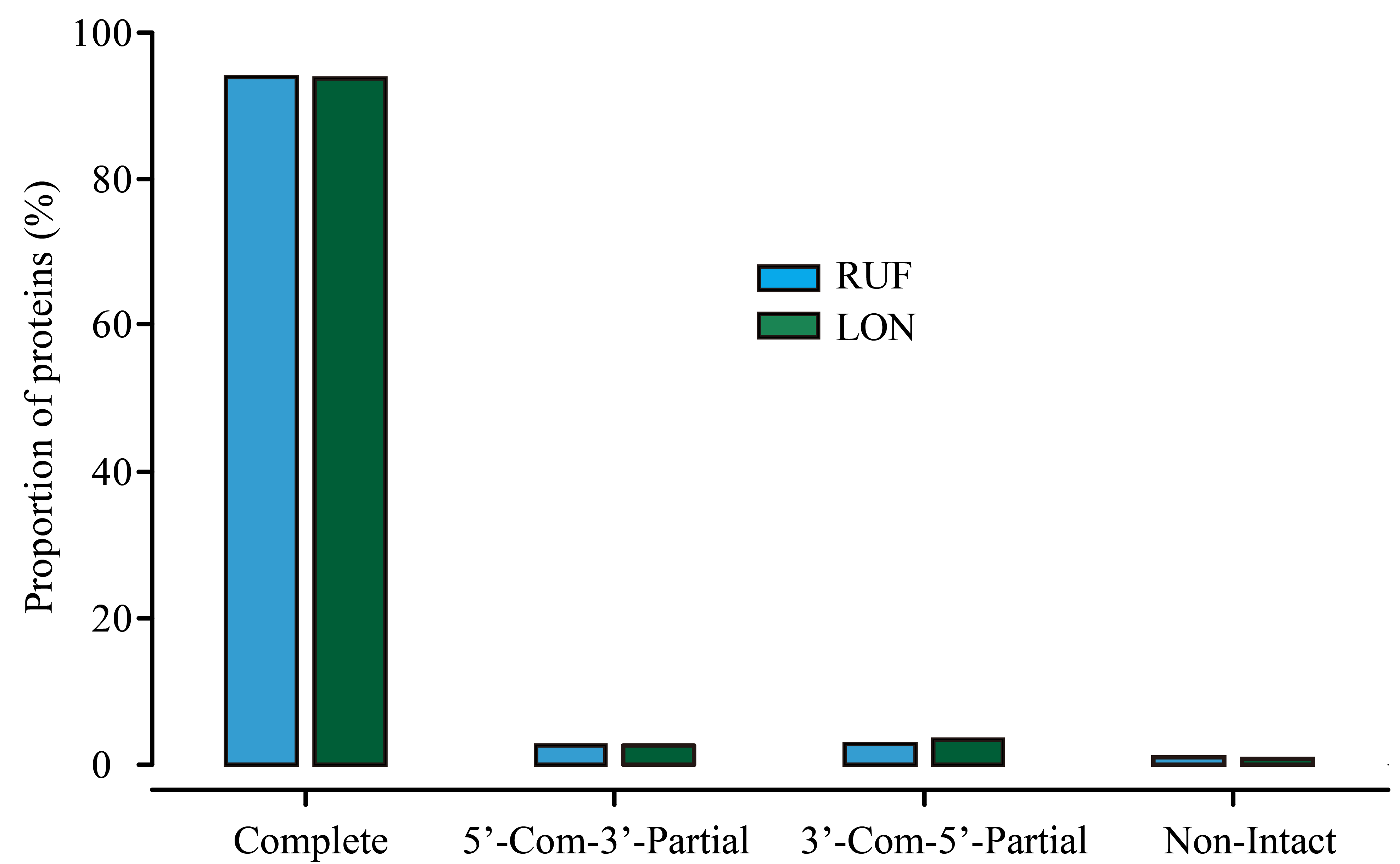


**Supplementary Fig. 6. Completeness validation of the predicted gene models of *O. rufipogon* and *O. longistaminata***. The complete gene models contain both start and stop codons. “Com” means “complete”, blue represents RUF, and green indicates LON.
