# Supplementary material for: Draft genomes of two outcrossing wild rice, Oryza rufipogon and O. longistaminata, reveal genomic features associated with mating‐system evolution
Source: Plant Direct. 2020 Jun 11;4(6):e00232. doi: 10.1002/pld3.232 (PMC7287411; doi:10.1002/pld3.232)
Supplement: Supplementary file 2 — Tables S1‐S16‐S18‐S19‐S24‐S26 [file PLD3-4-e00232-s002.docx]

**Supplementary Tables**

**Supplementary Table 1. Wild rice species sequenced in this study.**

| **Species** | **Geographical Origins** | **Accession No.** |
| --- | --- | --- |
| ***O. rufipogon*** | Yunnan Province, China | NA ^a^ |
| ***O. longistaminata*** | Botswana | 81967 ^b^ |

^a^ The germplasm was collected by L. Z. Gao.

^b^ Provided by the Genetic Resources Center (GRC), International Rice Research Institute (IRRI).

**Supplementary Table 2. Libraries and read statistics used for sequence assembly of** **two wild rice species.**

| **Species** | **Data Type** | **Insert Size (bp)** | | **Length (bp)** | **Clean Data (Gb)** | | **Sequence Coverage** |
| --- | --- | --- | --- | --- | --- | --- | --- |
| **RUF** | **Total** | | | | 174.85 | 450.65 | |
|  | **Paired-ends** | | | | 69.71 | 179.67 | |
|  |  | 180 | 100 | | 18.39 | 47.40 | |
|  |  | 300 | 35-100 | | 22.43 | 57.81 | |
|  |  | 500 | 100-120 | | 28.89 | 74.46 | |
|  | **Mate pair** | | | | 104.54 | 269.43 | |
|  |  | 2K | 60-100 | | 8.85 | 22.81 | |
|  |  | 4K | 75-100 | | 30.97 | 79.82 | |
|  |  | 6K | 75-100 | | 6.28 | 16.18 | |
|  |  | 8K | 40-100 | | 31.51 | 81.21 | |
|  |  | 20K | 100 | | 0.6 | 1.55 | |
|  |  | 40K | 100 | | 26.33 | 67.86 | |
|  | **454 data** | |  | | 0.6 | 1.55 | |
| **LON** | **Total** | | | | 176.09 | 449.22 | |
|  | **Paired-ends** | |  | | 71.31 | 181.92 | |
|  |  | 300 | 100-120 | | 13.21 | 33.70 | |
|  |  | 360 | 100-120 | | 49.74 | 126.89 | |
|  |  | 500 | 100-120 | | 8.36 | 21.33 | |
|  | **Mate pair** | |  | | 104.78 | 267.30 | |
|  |  | 2k | 35-100 | | 44.75 | 114.16 | |
|  |  | 4k | 40 | | 1.75 | 4.46 | |
|  |  | 5k | 100 | | 25.31 | 64.57 | |
|  |  | 6k | 40 | | 1.14 | 2.91 | |
|  |  | 8k | 70 | | 31.83 | 81.20 | |

**Supplementary Table 3. Genome size of *O. rufipogon* and *O. longistaminata* estimated by flow cytometry and *k*-mer analysis.** *O. sativa* (the estimated genome size = 389 Mb) was employed as a inner standard, and the conversion factor used was 1 pg = 978 Mb (Doležel *et al*. 2003).

| **Species** | **Estimate Size (Mb)** | | | |
| --- | --- | --- | --- | --- |
|  | **Flow Cytometry Estimate (Mb)** | **17 *K*-mer Estimated Length (Mb)** | **Previously Estimated Size** |  |
| ***O. rufipogon*** | 383.38 | 387.78 | 439 ^a^, 459 ^b^ |  |
| ***O. longistaminata*** | 362.90 | 392.00 | 389 ^a^ |  |

^a^ Uozu, Ikehashi *et al*. 1997;

^b^ Ammiraju, Luo *et al*. 2006.

**Supplementary Table 4. Assembly statistic of the two sequenced genomes.**

| **Assembly Features** | | **RUF** | **LON** |
| --- | --- | --- | --- |
| **Assembled Length (bp)** | | 441,412,135 | 332,077,902 |
|  | **Scaffold N50 (bp)** | 1,935,928 | 1,133,199 |
|  | **Scaffold N90 (bp)** | 420,811 | 304,339 |
|  | **Contig N50 (bp)** | 18,879 | 16,885 |
|  | **Contig N90 (bp)** | 4,751 | 5,103 |
|  | **Scaffold Number** | 1,019 | 1,575 |
|  | **Contig Number** | 39,860 | 31,900 |
|  | **Longest Scaffold (bp)** | 7,596,770 | 4,945,523 |
|  | **Longest Contig (bp)** | 232,066 | 134,867 |
| **>1Mb Scaf Length (bp)** | | 333,489,628 | 177,569,039 |
|  | **0.1 Mb – 1 Mb** | 97,399,193 | 145,579,227 |
|  | **10 Kb – 100 Kb** | 9,106,835 | 6,759,435 |
|  | **1 Kb – 10 Kb** | 1,392,122 | 2,064,879 |
|  | **1 bp – 1 Kb** | 24,357 | 105,322 |
| **Estimated Length (Mb)** | | 388 | 392 |
| **No gap Length (bp)** | | 371,661,267 | 297,870,486 |
|  | **Gap Number** | 38,841 | 30,325 |
|  | **Gap Length (bp)** | 69,750,868 | 34,207,416 |
| **GC Content (%)** | | 42.52 | 41.07 |

**Supplementary Table 5. Scaffold length constitutions of the two sequenced genomes.**

| **Species** | **Scaffold Length** | **Number** | **Scaffold Length (bp)** | **Average Length (bp)** | **Percentage (%)** |
| --- | --- | --- | --- | --- | --- |
| **RUF** | **>1 Kb** | 988 | 441,387,778 | 446,749 | 99.99% |
|  | **>10 Kb** | 651 | 439,995,656 | 675,877 | 99.68% |
|  | **>50 Kb** | 454 | 435,212,761 | 958,618 | 98.60% |
|  | **>100 Kb** | 391 | 430,888,821 | 1,102,017 | 97.62% |
|  | **>200 Kb** | 326 | 421,113,527 | 1,291,759 | 95.40% |
|  | **>300 Kb** | 279 | 409,477,601 | 1,467,662 | 92.77% |
|  | **>500 Kb** | 221 | 385,710,803 | 1,745,298 | 87.38% |
|  | **>800 Kb** | 164 | 350,430,992 | 2,136,774 | 79.39% |
|  | **>1 Mb** | 145 | 333,489,628 | 2,299,928 | 75.55% |
|  | **>2 Mb** | 63 | 212,329,955 | 3,370,317 | 48.10% |
|  | **>3 Mb** | 33 | 139,760,529 | 4,235,168 | 31.66% |
| **LON** | **>1 Kb** | 1,441 | 293,332,926 | 203,562 | 99.96 |
|  | **>10 Kb** | 614 | 291,234,947 | 474,324 | 99.25 |
|  | **>50 Kb** | 470 | 288,144,498 | 613,073 | 98.20 |
|  | **>100 Kb** | 419 | 284,459,217 | 678,900 | 96.94 |
|  | **>200 Kb** | 359 | 275,862,081 | 768,418 | 94.01 |
|  | **>300 Kb** | 291 | 259,114,977 | 890,429 | 88.30 |
|  | **>500 Kb** | 207 | 226,722,730 | 1,095,279 | 77.26 |
|  | **>800 Kb** | 122 | 172,981,379 | 1,417,880 | 58.95 |
|  | **>1 Mb** | 91 | 145,661,459 | 1,600,675 | 49.64 |
|  | **>2 Mb** | 18 | 43,782,408 | 2,432,356 | 14.92 |
|  | **>3 Mb** | 3 | 14,884,925 | 4,961,641 | 4.48 |

**Supplementary Table 6. Quality assessment of the genome assembly using DNA, protein, NGS short reads and EST datasets.**

| **Species** | **Evidences** | **Sources** | **Total** | **Aligned** | **Percentage (%)** |
| --- | --- | --- | --- | --- | --- |
| **RUF** | **DNA*** | NCBI (*O. rufipogon*) | 20,667 | 17,968 | 86.94 |
|  | **Protein**** | NCBI (*O. rufipogon*) | 3,762 | 2,424 | 64.43 |
|  |  | *O. sativa* ssp. *japonica* | 66,338 | 43,954 | 66.26 |
|  |  | *O. nivara* | 48,360 | 31,012 | 64.13 |
|  | **EST*** | Assembled in this study | 146,397 | 122,091 | 83.40 |
| **LON** | **DNA*** | NCBI (*O. longistaminata*) | 516 | 436 | 84.50 |
|  | **Protein**** | NCBI (*O. longistaminata*) | 740 | 489 | 66.08 |
|  |  | *O. sativa* ssp. *japonica* | 39,049 | 23,700 | 60.69 |
|  | **EST*** | Assembled in this study | 111,105 | 86,043 | 77.44 |

*Aligned using GMAP (version 2014-10-22), and the hits with identity ≥ 90% && coverage ≥ 90% are retained.

**Aligned using genBlastA (version 1.0.1), and the hits with identity ≥ 80% && coverage ≥ 90% are retained.

Note: The DNA and protein sequences were retrieved from NCBI database. The chloroplast and mitochondrion sequences were removed.

**Supplementary Table 7. Validation of the genome assembly using reads mapping.**

| **Species** | **Lanes** | **Insert Size (bp)** | **Read Length (bp)** | **Number of Reads Pairs** | **Number of Mapped Reads** | **Mapping Rate (%)** |
| --- | --- | --- | --- | --- | --- | --- |
| **RUF** | **PE01** | 180 | 100 | 167,866,394 | 157,918,634 | 94.07 |
|  | **PE02** | 240 | 100 | 42,928,086 | 40,637,285 | 94.66 |
|  | **PE03** | 340 | 75 | 72,077,852 | 68,601,066 | 95.18 |
|  | **PE04** | 340 | 84 | 61,463,706 | 58,215,027 | 94.71 |
|  | **PE05** | 360 | 75 | 25,140,454 | 23,931,139 | 95.19 |
|  | **PE06** | 370 | 100 | 86,983,764 | 82,780,207 | 95.17 |
|  | **PE07** | 430 | 118 | 8,342,596 | 7,848,284 | 94.07 |
|  | **PE08** | 460 | 100 | 109,183,088 | 102,486,501 | 93.87 |
|  | **Total** |  |  | 573,985,940 | 542,418,143 | 94.50 |
| **LON** | **PE01** | 300 | 121 | 78,062,078 | 66,229,594 | 84.84 |
|  | **PE02** | 300 | 121 | 64,829,362 | 55,776,899 | 86.04 |
|  | **PE03** | 500 | 121 | 66,833,698 | 57,314,073 | 85.76 |
|  | **PE04** | 500 | 121 | 25,204,130 | 21,118,678 | 83.79 |
|  | **Total** |  |  | 234,929,268 | 200,439,244 | 85.31 |

**Supplementary Table 8. Assessment of the assembly quality of *O. rufipogon* mapped to Nipponbare (MSU v7.0) genome.**

|  | **Nipponbare (MSU v7.0)** | | | | **The RUF Genome Mapped to the Repeat Sequence-free SAT Genome^c^** | | | | **The LON Genome Mapped to the Repeat Sequence-free SAT Genome^c^** | | |
| --- | --- | --- | --- | --- | --- | --- | --- | --- | --- | --- | --- |
|  | **Sequence Length (bp)** | **Remained Length^a^ (bp)** | | **Remained Percentage^b^ (%)** | **Mapped Length (bp)** | **Mapped Percentage (%)** | | **Sequence Similarity^c^ (%)** | **Mapped Length (bp)** | **Mapped Percentage (%)** | **Sequence Similarity^c^ (%)** |
| **Chr01** | 43270923 | 27306172 | 63.11 | | 23073774 | 84.50 | | 97.19 | 18660152 | 68.34 | 94.95 |
| **Chr02** | 35937250 | 22687573 | 52.84 | | 19575613 | 86.28 | | 97.32 | 15771082 | 69.51 | 94.86 |
| **Chr03** | 36413819 | 23693595 | 53.12 | | 20645601 | 87.14 | | 97.34 | 16658042 | 70.31 | 94.98 |
| **Chr04** | 35502694 | 19372428 | 52.83 | | 15968696 | 82.43 | | 97.12 | 12668363 | 65.39 | 94.76 |
| **Chr05** | 29958434 | 16885298 | 63.13 | | 14411647 | 85.35 | | 97.00 | 11309679 | 66.98 | 94.56 |
| **Chr06** | 31248787 | 17787653 | 65.07 | | 14645648 | 82.34 | | 96.83 | 11808316 | 66.38 | 94.83 |
| **Chr07** | 29697621 | 16516279 | 54.57 | | 13567913 | 82.15 | | 96.81 | 10832541 | 65.59 | 94.82 |
| **Chr08** | 28443022 | 15586018 | 56.36 | | 12927950 | 82.95 | | 97.05 | 10341283 | 66.35 | 94.70 |
| **Chr09** | 23012720 | 12709022 | 56.92 | | 10569811 | 83.17 | | 97.01 | 8278415 | 65.14 | 94.74 |
| **Chr10** | 23207287 | 12261737 | 55.61 | | 10164391 | 82.90 | | 96.88 | 8146793 | 66.44 | 94.87 |
| **Chr11** | 29021106 | 15415670 | 54.8 | | 11985859 | 77.75 | | 96.59 | 9260643 | 60.07 | 94.49 |
| **Chr12** | 27531856 | 14545811 | 55.23 | | 11076586 | 76.15 | | 96.44 | 9101936 | 62.57 | 94.69 |
| **Total** | 373245519 | 214767256 | 57.54 | | 178613489 | | 83.17 | 97.02 | 142837245 | 66.51 | 94.79 |

^a^ Remained length of the SAT genome by removing repeat sequences using RepeatMasker; ^b^ Remained percentage of the SAT genome by removing repeat sequences using RepeatMasker; ^c^ The RUF genome mapped to the repeat sequence-free SAT genome using Mummer.

|  |
| --- |

**Supplementary Table 9. Comparisons of the assembled scaffolds of *O. rufipogon* against the SMRT contigs.**

| **Scaffolds** | **SMRT** | **Overlapping Length (bp)** | **Overall Similarity** | **Similarity (repeat sequence-free)** |
| --- | --- | --- | --- | --- |
| Scaffold149 | utg2125 | 444,105 | 98.92% | 99.64% |
| Scaffold149 | utg2654 | 614,545 | 99.08% | 99.54% |
| Scaffold225 | utg93 | 680,863 | 98.90% | 99.54% |
| Scaffold281 | utg512 | 423,616 | 98.89% | 99.57% |
| Scaffold334 | utg212 | 385,374 | 98.86% | 99.58% |

**Supplementary Table 10. RNA_Seq data of two wild *Oryza* species from four tissues using the Illumina Hiseq2000 platform.**

| **Species** | **Platform** | **Tissues** | **Read Length (bp)** | **Number of Pair Reads*** | **Clean Data (Gb)** |
| --- | --- | --- | --- | --- | --- |
| **RUF** | **Illumina** | **30-d-roots**  **30-d-shoots**  **Panicles at booting stage**  **Flag leaves at booting stage**  **Total** | 100  100  100  100 | ~39 M  ~34 M  ~48 M  ~29 M  ~150 M | ~7.86  ~6.71  ~9.52  ~5.70  ~29.79 |
| **RUF** | **Roche 454** | **30-d-roots**  **30-d-shoots**  **Panicles at booting stage**  **Flag leaves at booting stage**  **Total** | 272  303  277  299 | ~0.16  ~0.25  ~0.12  ~0.24  ~0.76 | ~0.043  ~0.076  ~0.033  ~0.071  ~0.223 |
| **LON** |  | **30-d-roots**  **30-d-shoots**  **Panicles at booting stage**  **Flag leaves at booting stage**  **Total** | 100  100  100  100 | ~33 M  ~41 M  ~29 M  ~37 M  ~140 M | ~6.36  ~7.82  ~5.51  ~7.04  ~26.73 |

* M indicates million.

**Supplementary Table 11. Assembly statistics of the transcriptomes from two wild *Oryza* species.**

| **Species** | **RUF** | | **LON** |
| --- | --- | --- | --- |
| **Platforms** | **Illumina*** | **454**** | **Illumina*** |
| **Total number of transcripts** | 109,000 | 33,496 | 111,105 |
| **Total length (bp)** | 112,162,383 | 18,905,412 | 74,561,481 |
| **N10 (bp)** | 3,041 | 1,252 | 3,378 |
| **N20 (bp)** | 2,269 | 955 | 2,477 |
| **N30 (bp)** | 1,832 | 776 | 1,912 |
| **N40 (bp)** | 1,492 | 662 | 1,461 |
| **N50 (bp)** | 1,193 | 582 | 1,064 |
| **Average length (bp)** | 766 | 564 | 671 |
| **GC content (%)** | 48.81 | 44.19 | 47.46 |

* Transcriptome was assembled using Trinity (version 2.2.0) with default parameters.

** Transcriptome was assembled using MIRA (Version 4.0.2) with default parameters.

**Supplementary Table 12. Prediction of protein-coding genes in the two wild *Oryza* species compared with Nipponbare (MSU v7.0).**

|  | ***O. rufipogon*** | ***O. longistaminata*** | **Nipponbare (MSU v7.0)** |
| --- | --- | --- | --- |
| **# Total number of predicted genes** | 52,997 | 40,014 | 55,986 |
| **# Total number of gene models** | 61,366 | 45,812 | 66,338 |
| **Average gene length (bp)** | 2,627 | 2,769 | 2,853 |
| **Average CDS length (bp)** | 1,004 | 978 | 1,332 |
| **Average CDS GC ratio (%)** | 53.9 | 50.9 | 53.0 |
| **Average exons per gene** | 4.2 | 4.6 | 4.7 |
| **TE-related genes** | 2,994 | 2,767 | 16,941 |
| **TE-related gene models** | 3,780 | 3,212 | 17,272 |

**Supplementary Table 13. Validation of gene model prediction of two wild *Oryza* species using protein and RNA sequencing data.**

|  | **RUF** | | **LON** | |
| --- | --- | --- | --- | --- |
|  | **Number** | **Percentage (%)** | **Number** | **Percentage (%)** |
| Total predicted genes | 52,997 | 100.0 | 40,014 | 100.0 |
| Protein supported ^a^ | 39,600 | 74.7 | 26,059 | 65.1 |
| RNA-Seq supported | 31,553 | 59.5 | 27,061 | 67.6 |
| Protein and RNA-Seq supported | 23,541 | 44.4 | 20,460 | 51.1 |
| Protein or RNA-Seq supported | 47,612 | 89.8 | 32,660 | 81.6 |

^a^ Protein supported criterion: identity ≥ 30%; coverage ≥ 90%;

**Supplementary Table 14. Summary of the annotated repeat sequences in two wild *Oryza* species.**

|  | ***O. rufipogon*** | | ***O. longistaminata*** | | ***Nipponbare***  **(MSU v7.0)** | | |  |
| --- | --- | --- | --- | --- | --- | --- | --- | --- |
|  | **Length**  **(Mp)** | **Percentage**  **(%) ^b^** | **Length**  **(Mp)** | **Percentage**  **(%) ^b^** | | **Length**  **(Mp)** | **Percentage**  **(%) ^b^** | |
| **Total Repeats** | 167.91 | 45.18% | 109.28 | 36.69% | | 194.8 | 50.97% | |
| **DNA transposons** | 92.78 | 24.96% | 64.10 | 21.52% | | 89.84 | 23.51% | |
| ***CACTA*** | 8.03 | 2.16% | 4.49 | 1.51% | | 12.35 | 3.23% | |
| ***PIF/Harbinger*** | 14.78 | 3.98% | 11.77 | 3.95% | | 9.66 | 2.53% | |
| ***hAT*** | 4.86 | 1.31% | 3.56 | 1.19% | | 3.61 | 0.94% | |
| ***MULE*** | 41.67 | 11.21% | 19.45 | 6.53% | | 47.27 | 12.37% | |
| ***Tc1/Mariner*** | 10.96 | 2.95% | 8.59 | 2.88% | | 5.79 | 1.52% | |
| **RC*/Helitron*** | 10.57 | 2.84% | 14.32 | 4.81% | | 8.78 | 2.30% | |
| **Other** | 1.92 | 0.52% | 1.93 | 0.65% | | 2.38 | 0.62% | |
| **RNA transposons** | 64.90 | 17.46% | 38.60 | 12.96% | | 96.19 | 25.17% | |
| **Non-LTR retrotransposons** | 8.17 | 2.20% | 6.84 | 2.29% | | 7.3 | 1.91% | |
| **LINE** | 6.33 | 1.70% | 5.46 | 1.83% | | 5.95 | 1.56% | |
| **SINE** | 1.84 | 0.49% | 1.37 | 0.46% | | 1.36 | 0.36% | |
| **LTR retrotransposons** | 56.73 | 15.26% | 31.77 | 10.66% | | 88.89 | 23.26% | |
| **Ty1-*Copia*** | 13.80 | 3.71% | 6.22 | 2.09% | | 19.81 | 5.18% | |
| **Ty3-*Gypsy*** | 38.32 | 10.31% | 22.29 | 7.48% | | 60.37 | 15.80% | |
| **Other ^a^** | 4.61 | 1.24% | 3.26 | 1.09% | | 8.71 | 2.28% | |
| **Simple Repeats** | 4.30 | 1.16% | 2.90 | 0.97% | | 4.11 | 1.07% | |
| **Other Repeats** | 5.93 | 1.59% | 3.68 | 1.23% | | 4.66 | 1.22% | |

**a.** LTR retrotransposons that cannot be classified into either *Gypsy* or *Copia* super-families;

**b.** Percentage (%) to contig length without N, 371,674,463 for RUF and 297,888,484 for LON, respectively.

**Supplementary Table 15. Summary of types and number of simple sequence repeats in the sequenced genomes.**

| **Species** | **Types** | **Total** | | | **The most plentiful types** | |
| --- | --- | --- | --- | --- | --- | --- |
|  |  | **Subtype** | **N ≥ 3 &**  **L ≥ 12** | **L ≥ 20** | **Types** | **L ≥ 12** |
| **RUF** | **Monomer** | 2 | 16,810 | 964 | A/T | 14,956 |
|  | **Dimer** | 4 | 33,281 | 7,282 | AG/CT | 15,980 |
|  | **Trimer** | 10 | 81,052 | 5,898 | CCG/CGG | 31,400 |
|  | **Tetramer** | 33 | 55,679 | 3,150 | AAAT/ATTT | 5,773 |
|  | **Pentamer** | 102 | 17,264 | 2,679 | AAAAT/ATTTT | 1,636 |
|  | **Hexamer** | 330 | 10,251 | 10,251 | ACGGCG/CCGTCG | 516 |
| **Total** | | 481 | 214,337 | 30,224 |  |  |
| **LON** | **Monomer** | 2 | 19,229 | 531 | A/T | 18,393 |
|  | **Dimer** | 4 | 19,863 | 3,999 | AG/CT | 10,049 |
|  | **Trimer** | 10 | 84,806 | 3,423 | CCG/CGG | 15,244 |
|  | **Tetramer** | 33 | 42,114 | 2,146 | AAAT/ATTT | 4,523 |
|  | **Pentamer** | 102 | 12,039 | 1,837 | AAAAT/ATTTT | 1,293 |
|  | **Hexamer** | 325 | 6,722 | 6,722 | AAAAAG/CTTTTT | 336 |
| **Total** | | 476 | 184,773 | 18,658 |  |  |

**Supplementary Table 16. Summary of non-coding RNA genes.**

| **Species** | **Type** | **Number** | **Average Length (bp)** | **Total Length (bp)** | **% of Genome** |
| --- | --- | --- | --- | --- | --- |
| **RUF** | **tRNA** | 733 | 74 | 54,232 | 0.0115% |
|  | **rRNA (8S)** | 57 | 114 | 6,494 | 0.0014% |
|  | **rRNA (18S)** | 4 | 1574 | 6,294 | 0.0013% |
|  | **rRNA (28S)** | 3 | 4434 | 13,301 | 0.0028% |
|  | **SnoRNA** | 284 | 97 | 27,659 | 0.0059% |
|  | **snRNA** | 146 | 144 | 21,012 | 0.0044% |
|  | **miRNA** | 271 | 130 | 35,150 | 0.0074% |
| **LON** | **tRNA** | 636 | 74 | 47,272 | 0.0143% |
|  | **rRNA(8s)** | 1 | 137 | 137 | 0.0000% |
|  | **rRNA(28s)** | 1 | 7,360 | 7,360 | 0.0022% |
|  | **SnoRNA** | 247 | 94 | 23,293 | 0.0070% |
|  | **snRNA** | 124 | 142 | 17,556 | 0.0053% |
|  | **miRNA** | 340 | 130 | 44,017 | 0.0133% |

**Supplementary Table 18. Statistic of the proteins for the nine *Oryza* genomes using OrthoMCL.**

| **Species** | **Total** | **TE-related Proteins*** | **The Analyzed Proteins** | **Percentage**  **(%)** |
| --- | --- | --- | --- | --- |
| **SAT** | 55,986 | 16,386 | 39,600 | 70.73 |
| **RUF** | 52,997 | 1,964 | 51,033 | 96.29 |
| **NIV** | 41,490 | 1,867 | 39,623 | 95.50 |
| **GLA** | 41,479 | 1,681 | 39,798 | 95.95 |
| **BAR** | 42,284 | 1,779 | 40,505 | 95.79 |
| **GLU** | 41,605 | 1,741 | 39,864 | 95.82 |
| **LON** | 40,014 | 1,589 | 38,425 | 96.03 |
| **MER** | 39,106 | 1,616 | 37,490 | 95.87 |
| **PUN** | 31,762 | 816 | 30,946 | 97.43 |
| **Total** | 386,723 | 19,439 | 357,284 | 92.39 |

***** Gene models were aligned with MSU *Oryza* Repeat Database, and matches with coverage > 40% and E-value < 1e-5 were annotated as TE-related genes.

**Supplementary Table 19.** **Summary of the gene families identified among the eight AA- *Oryza* genomes using *O. punctata* (BB- genome) as outgroup.**

| **Species** | **Gene family** | **Genes within gene family** | **Lineage-specific gene family** | **Genes within lineage-specific gene family** |
| --- | --- | --- | --- | --- |
| **SAT** | 27,102 | 35,571 | 89 | 234 |
| **RUF** | 27,039 | 39,757 | 650 | 1,459 |
| **NIV** | 28,377 | 34,740 | 47 | 98 |
| **GLA** | 30,558 | 37,055 | 33 | 73 |
| **BAR** | 29,167 | 35,555 | 63 | 131 |
| **GLU** | 28,824 | 35,360 | 54 | 111 |
| **LON** | 20,356 | 28,183 | 313 | 652 |
| **MER** | 25,378 | 31,701 | 122 | 264 |
| **PUN** | 19,705 | 26,179 | 166 | 389 |
| **Total** | 236,506 | 304,101 | 1,537 | 3,411 |

**Supplementary Table 24.** **Number of NBS-LRR genes identified in the nine *Oryza* genomes.**

| **Type** | **SAT** | **RUF** | **NIV** | **GLA** | **BAR** | **GLU** | **LON** | **MER** | **PUN** |
| --- | --- | --- | --- | --- | --- | --- | --- | --- | --- |
| **CC-NBS** | 56 | 147 | 64 | 63 | 61 | 43 | 94 | 53 | 39 |
| **CC-NBS-LRR** | 252 | 241 | 133 | 121 | 136 | 129 | 213 | 116 | 170 |
| **NBS-LRR** | 227 | 284 | 178 | 158 | 161 | 125 | 301 | 136 | 148 |
| **TIR-NBS** | 1 | 1 | 1 | 1 | 1 | 1 | 1 | 1 | 1 |
| **NBS** | 95 | 172 | 113 | 107 | 117 | 94 | 159 | 110 | 68 |
| **Total** | 631 | 845 | 489 | 450 | 476 | 392 | 768 | 416 | 426 |

**Supplementary Table 25.** **Coverage of orthologous genomic regions in the eight AA-genome *Oryza* species.**

| **Species** | **Size of genome (bp)** | **Length of aligned region (bp)** | **Coverage** | **Gene number** | **The average gene number per block** |
| --- | --- | --- | --- | --- | --- |
| **SAT**  **RUF**  **NIV**  **GLA**  **BAR**  **GLU**  **LON**  **MER** | 374,470,183  472,702,210  379,501,719  350,231,320  346,015,512  338,711,595  332,077,902  347,092,393 | 195,653,459  189,175,779  179,975,073  184,166,392  180,021,270  163,295,623  133,371,502  141,674,922 | 52.25%  40.02%  47.42%  52.58%  52.03%  48.21%  40.16%  40.82% | 39,045  52,997  41,490  41,479  42,283  41,605  40,014  39,106 | 4.47  6.06  4.74  4.74  4.84  4.76  4.58  4.47 |

**Supplementary Table 26.** **Species-specific rapid evolving regions in CNSs in the eight AA-genome *Oryza* species.**

| **Species** | **Number** | **Total Length (bp)** | **Total Mutation (bp)** | **Mutation frequency (%)** |
| --- | --- | --- | --- | --- |
| **SAT** | 2,022 | 2,278,873 | 69,763 | 3.06% |
| **RUF** | 1,905 | 2,144,263 | 81,199 | 3.79% |
| **NIV** | 1,955 | 2,153,624 | 75,913 | 3.52% |
| **GLA** | 2,012 | 2,252,665 | 73,043 | 3.24% |
| **BAR** | 1,998 | 2,238,846 | 73,829 | 3.30% |
| **GLU** | 1,889 | 2,131,795 | 84,571 | 3.97% |
| **LON** | 2,086 | 2,294,793 | 130,269 | 5.68% |
| **MER** | 2,132 | 2,345,804 | 153,656 | 6.55% |
